# Overall structure of fully assembled cyanobacterial KaiABC circadian clock complex by an integrated experimental-computational approach

**DOI:** 10.1038/s42003-022-03143-z

**Published:** 2022-03-10

**Authors:** Yasuhiro Yunoki, Atsushi Matsumoto, Ken Morishima, Anne Martel, Lionel Porcar, Nobuhiro Sato, Rina Yogo, Taiki Tominaga, Rintaro Inoue, Maho Yagi-Utsumi, Aya Okuda, Masahiro Shimizu, Reiko Urade, Kazuki Terauchi, Hidetoshi Kono, Hirokazu Yagi, Koichi Kato, Masaaki Sugiyama

**Affiliations:** 1 Exploratory Research Center on Life and Living Systems (ExCELLS) and Institute for Molecular Science (IMS), National Institutes of Natural Sciences, 5-1 Higashiyama, Myodaiji-cho, Okazaki, 444-8787 Japan; 2 Graduate School of Pharmaceutical Sciences, Nagoya City University, 3-1 Tanabe-dori, Mizuhoku, Nagoya, 467-8603 Japan; 3 Institute for Quantum Life Science, National Institutes for Quantum Science and Technology (QST), Umemidai, Kizu, Kyoto 619-0215 Japan; 4 Institute for Integrated Radiation and Nuclear Science, Kyoto University, 2-1010 Asashironishi, Kumatori, Sennan-gun, Osaka 590-0494 Japan; 5 Institut Laue-Langevin, 71, avenue des martyrs, 38042 Grenoble, France; 6 Neutron Science and Technology Center, Comprehensive Research Organization for Science and Society (CROSS), Tokai, Ibaraki, 319-1106 Japan; 7 Graduate School of Life Sciences, Ritsumeikan University, 1-1-1 Noji-higashi, Kusatsu, Shiga, 525-8577 Japan; 8 Present Address: Institute for Integrated Radiation and Nuclear Science, Kyoto University, 2-1010 Asashironishi, Kumatori, Sennan-gun, Osaka 590-0494 Japan; 9 Present Address: Biomedical Research Centre, School of Biomedical Engineering, The University of British Columbia, 2222 Health Sciences Mall, Vancouver, BC V6T 1Z3 Canada

**Keywords:** SAXS, Computational biophysics

## Abstract

In the cyanobacterial circadian clock system, KaiA, KaiB and KaiC periodically assemble into a large complex. Here we determined the overall structure of their fully assembled complex by integrating experimental and computational approaches. Small-angle X-ray and inverse contrast matching small-angle neutron scatterings coupled with size-exclusion chromatography provided constraints to highlight the spatial arrangements of the N-terminal domains of KaiA, which were not resolved in the previous structural analyses. Computationally built 20 million structural models of the complex were screened out utilizing the constrains and then subjected to molecular dynamics simulations to examine their stabilities. The final model suggests that, despite large fluctuation of the KaiA N-terminal domains, their preferential positionings mask the hydrophobic surface of the KaiA C-terminal domains, hindering additional KaiA-KaiC interactions. Thus, our integrative approach provides a useful tool to resolve large complex structures harboring dynamically fluctuating domains.

## Abstract (web summary)

The revealed full KaiA_12_B_6_C_6_ complex is assembled including the dynamic and asynchronous KaiA N-terminal domains that have been missing in cryo-EM structures.

## Introduction

Homeostatic activities of biological systems are regulated through dynamically concerted assembly and disassembly of biomolecules^1–5^. This is best exemplified by the circadian clock in cyanobacteria (Kai-clock), which is constituted of three proteins, KaiA, KaiB, and KaiC. These proteins undergo an association–dissociation cycle coupled with phosphorylation–dephosphorylation oscillation of KaiC in the presence of adenosine triphosphate (ATP)^6,7^. During the circadian cycle, the Kai-clock system generates three forms of complex, two binary KaiAC and KaiBC complexes and one ternary KaiABC complex, at specific clock phases^8,9^.

KaiA consists of N-terminal domain (residues 1–161, referred to as _N_A), canonical linker (residues 162–181), and C-terminal domain (residues 182–284, referred to as _C_A) and forms a homodimer (A_2_) through cA^10,11^. KaiB assumes a single thioredoxin domain and forms a homotetramer (B_4_)^12,13^. KaiC consists of two domains (CI and CII) and forms a homohexamer (C_6_) with a double doughnut-like shape^14–16^. The KaiC hexamer can interact with one A_2_ dimer through the C-terminal tails of CII domains, giving rise to the A_2_C_6_ complex^17–19^. The KaiC hexamer can also bind six KaiB molecules, which are arranged in a hexameric ring on the top of the CI domains of C_6_, forming the B_6_C_6_ complex^8,20,21^. Regarding the ternary KaiA-KaiB-KaiC complex (ABC complex), not only supramolecular architecture but also stoichiometry in the complex have been controversial for a long time^22–24^. Recently, a cryo-EM study revealed the structure of ABC complex (more precisely, A_12_B_6_C_6_ complex) in which six A_2_ dimers were captured onto the KaiB ring in the B_6_C_6_ subcomplex^25,26^. In that structure, each A_2_ interacts with one KaiB protomer through one of two dimerized _C_A domains. Hereafter, _C_A domains will be termed _C1_A and _C2_A depending if it is bound or unbound to KaiB, respectively. In total, 6 _C1_A and 6 _C2_A domains were visualized as a ring structure like an Elizabethan collar on the top of B_6_C_6_ subcomplex. In contrast, the _N_A domains were missing in the cryo-EM structure, suggesting that the _N_A domains could dynamically fluctuate in the A_12_B_6_C_6_ complex. Accordingly, the overall structure of A_12_B_6_C_6_ complex still remained to be completely solved.

The ABC complex is critical in switching from positive to negative feedback in both KaiC phosphorylation and complex formation cycles^20,27,28^. In addition, previously reported mutational studies indicated that the _N_A domain is essential for generation of circadian rhythm^29,30^. Therefore, it is crucially important to solve the overall structure of A_12_B_6_C_6_ complex, including the locations of _N_A domains.

Small-angle X-ray and neutron scattering (SAXS and SANS) provide overall structural information of supramolecular complex in solution^31–33^ and can potentially be used for investigating a dynamic structure by combination with computational analysis^34–36^. However, there are three issues to be addressed in elucidating the structure of A_12_B_6_C_6_ complex with small-angle scattering. The first is how to eliminate contributions to scattering from undesirable components. The A_12_B_6_C_6_ complex stably exists only in a solution mixture with over-saturation of A_2_, B_4_, and B_6_C_6_ complex. This means that the sample solution of A_12_B_6_C_6_ complex inevitably includes non-integrated A_2_, B_4_, and/or B_6_C_6_ complex and their aggregates as undesirable components, which interfere with SAXS measurements. To observe SAXS only from the A_12_B_6_C_6_ complex in the multi-component solution, we utilized SAXS coupled with size-exclusion chromatograph (SEC-SAXS)^37–39^.

The second issue is how to selectively observe the scattering originating from the components of interest in a complex. In the previous works, the structures and dynamics of multi-domain proteins and protein complexes were investigated with SAXS and coarse-grained molecular dynamics (CG-MD) simulations^40–42^. However, a single SAXS profile is not enough to analyze the structure of the large A_12_B_6_C_6_ complex with the fluctuating _N_A domains and then it is required to edit scattering data focusing on the KaiA protomers in question. For this purpose, we applied inverse contrast-matching SANS (iCM-SANS), which enables selective observation of hydrogenated component(s) within a biomacromolecular complex consisting of hydrogenated and deuterated components by taking advantage of the isotope effect of hydrogen in neutron scattering^4,21,33,43–46^. When we measured SANS of the A_12_B_6_C_6_ complex consisting of hydrogenated KaiA (hA), 75%-deuterated forms of KaiB and KaiC (dB and dC) in 100% D_2_O buffer, we selectively observe the hA protomers in hA_12_dB_6_dC_6_ complex. However, our sample solution also included hA_2_, dB_4_, dB_6_dC_6_, and their aggregates. To overcome such problems, we recently developed a method based on the combined use of SEC-SANS^47,48^ with iCM-SANS (SEC-iCM-SANS)^49^.

The third issue is how to build a three-dimensional structural model and characterize conformational dynamics of the large complex. To address this issue, we developed a method combining computational and experimental approaches. A vast array of computational models of the A_12_B_6_C_6_ complex were generated based on the cryo-EM and X-ray crystallographic structures and subjected to screening based on the SEC-SAXS and SEC-iCM-SANS data. Eventually, selected models were verified through molecular dynamics simulations.

By overcoming these challenges with the state-of-the-art solution scattering techniques, SEC-SAXS and SEC-iCM-SANS, in conjunction with the computational approach, the present study successfully provided information on the overall structure of A_12_B_6_C_6_ complex, highlighting spatial arrangements of the _N_A domains.

## Results

### Oligomeric state of the ABC complex

On the dephosphorylation process, KaiC hexamer interacts with six KaiBs and six KaiA dimers, thus forms the A_12_B_6_C_6_ complex (Supplementary Fig. 1a)^25,26^. We established a preparation method of the A_12_B_6_C_6_ complex under over-saturation conditions of A_2_, B_4_, and B_6_C_6_, thereby overcoming the instability of the ternary complex (see Materials and methods, Supplementary Figs. 1b, 2a). The sample was subsequently subjected to analytical ultracentrifugation (AUC), which confirmed that the major component (p4) was the A_12_B_6_C_6_ complex (Supplementary Fig. 2b and Supplementary Tables 1 and 2). The AUC profile also indicated, however, the presence of minor components, corresponding to the peaks, p1, p2, p3, and p5, in the sample solution. Because these minor components deteriorated the scattering data^50^, as indicated in the last column (contribution ratio in the forward scattering intensity, *t*) of Supplementary Table 2, it was essential to exclude them from the solution.

### SEC-SAXS of A_12_B_6_C_6_ complex

**Fig. 1:**
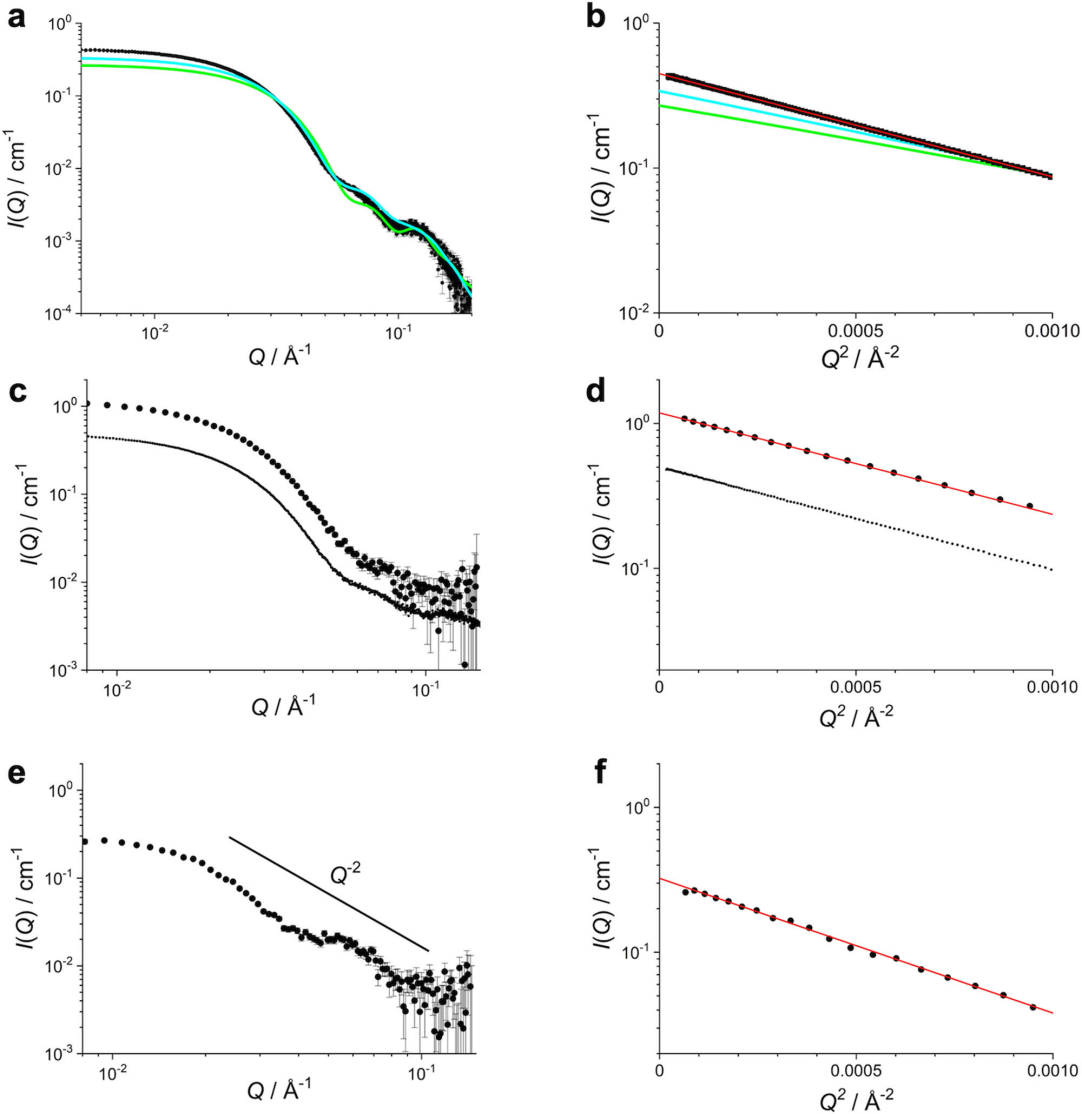
Scattering profiles and their Guinier plots of the A_12_B_6_C_6_ complex. **a** SAXS profiles and **b** their Guinier plots. Black circles show the SEC-SAXS profile and a green line does the SAXS profile calculated from the cryo-EM structure (χ^2^ = 1195)^5^. A cyan line expresses the SAXS profile of the overall A_12_B_6_C_6_ model (χ^2^ = 366). In this model, the missing _N_A domains were supplemented by superimposing six A_2_ dimers onto the cryo-EM structure. **c** SANS profiles of hA_12_hB_6_hC_6_ complex and **d** its Guinier plots. Small dots are the SAXS profiles and its Guinier plot for the reference. **e** SANS profiles of hA_12_dB_6_dC_6_ complex and **f** its Guinier plots. A straight line in panel **e** indicates *I*(*Q*) ~ *Q*^-2^. Red lines in panels **b**, **d**, and **f** show the results of the least-square fitting for the experimental data with Guinier formula. Error bars represent standard deviation of the mean.

SEC-SAXS enables the determination of the SAXS profile of the target molecular species in a multi-component mixture, by immediate measurement after its isolation by the SEC. Using this technique, we could separate the A_12_B_6_C_6_ complex from the minor components (Supplementary Fig. 2c). Figure 1a, b shows the SAXS profile corresponding to the A_12_B_6_C_6_ complex and its Guinier plot. The radius of gyration, ${R}_{{{{{{\rm{g}}}}}}}$, was calculated to be 69.5 ± 0.2 Å. We also calculated the SAXS profile of the A_12_B_6_C_6_ complex structure solved by cryo-EM^25^, which is depicted as a green line in Fig. 1a, b and indicates a ${R}_{{{{{{\rm{g}}}}}}}$ of 55.5 Å. The deviation of the SAXS profile (χ^2^ = 1195, defined as eq. (S1) in Supplementary note 1) from our experimental profile and the smaller ${R}_{{{{{{\rm{g}}}}}}}$ were ascribed to the lack of _N_A domains in the cryo-EM structure. An overall structural model of the A_12_B_6_C_6_ complex, by superimposing the full-length KaiA dimers onto the cryo-EM structure (see the procedure in Supplementary note 2) yielded the cyan line in Fig. 1a, b. The scattering profile became closer to the experimental one but still deviated largely (χ^2^ = 366). The calculated ${R}_{{{{{{\rm{g}}}}}}}$ was 62.2 Å, still smaller than that derived from the SEC-SAXS experimental value. This discrepancy is presumably due to dislocation of the _N_A domains in the A_12_B_6_C_6_ complex in solution.

### SEC-iCM-SANS of A_12_B_6_C_6_ complex

The iCM-SANS technique enables selective observation of hydrogenated components in a complex consisting of hydrogenated and 75%-deuterated components in 100% D_2_O solution (Supplementary Fig. 3a). We prepared the A_12_B_6_C_6_ complex with hydrogenated KaiA (hA), 75%-deuterated forms of KaiB and KaiC (dB and dC) (designated as hA_12_dB_6_dC_6_ complex), utilizing our established method (Supplementary Fig. 3b and see Supplementary note 3). The sample solution inevitably includes undesirable components, hA_2_, dB_4_, dB_6_dC_6_-complex and their aggregates. Consequently, we conducted SEC-iCM-SANS, which provided the scattering profile of hA protomers in the hA_12_dB_6_dC_6_ complex in the multi-component solution (Supplementary Fig. 3b).

Figure 1c, d shows a SANS profile of hA_12_hB_6_hC_6_ complex in D_2_O solution and its Guinier plot after SEC operation (Supplementary Fig. 2d). The SEC-iCM-SANS profile of fully hydrogenated hA_12_hB_6_hC_6_ complex and its ${R}_{{{{{{\rm{g}}}}}}}$ of 69.9 ± 0.4 Å well agreed with those obtained by the SEC-SAXS measurement of A_12_B_6_C_6_ complex (dotted lines in Fig. 1c, d) as expected. In contrast, the SEC-iCM-SANS profile of hA_12_dB_6_dC_6_ complex in D_2_O solution (Fig. 1e and Supplementary Fig. 2e) and its Guinier plot (Fig. 1f) were drastically different from those of hA_12_hB_6_hC_6_ complex (Fig. 1c, d). The scattering profile decreased with *Q*^−2^, indicating that a scatterer was a disk-like shape based on the classical interruption for a SAS profile^31^. This observation suggested that the six KaiA dimers were arranged in a doughnut-like shape on top of the B_6_C_6_ subcomplex as reported in the cryo-EM study. In addition, its larger ${R}_{{{{{{\rm{g}}}}}}}$ of 78.1 ± 1.0 Å supported this doughnut-like arrangement.

### A_12_B_6_C_6_ complex modeling

**Fig. 2:**
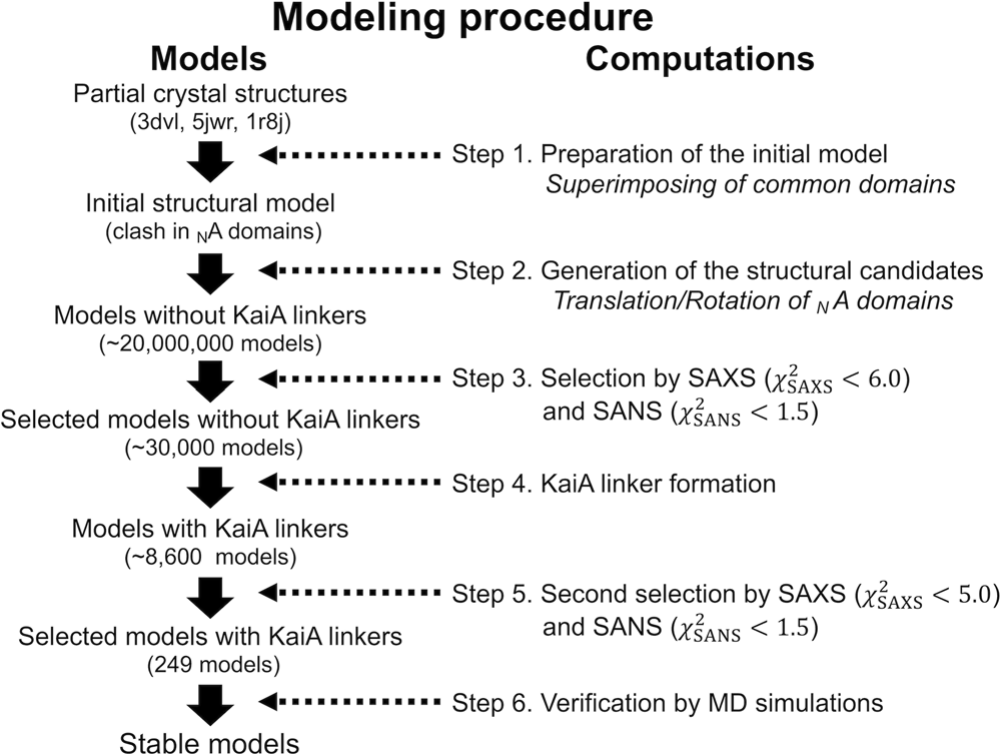
Modeling procedure of the overall structure of A_12_B_6_C_6_ complex. Left column denotes the structural models and right one does step-by-step computational process.

We computationally built three-dimensional models of the overall structure of A_12_B_6_C_6_ complex, which reproduced the SAXS and iCM-SANS profiles. The models obtained were further examined and screened through MD simulations from a viewpoint of stability. Our modeling procedure is described below (Fig. 2).

#### Step 1: Preparation of the initial model

Since the cryo-EM structure (PDB:5n8y) lacks the side-chain information, we built an initial model with crystal structures providing them. There are currently three crystal structures available that can be used to build the structure model of A_12_B_6_C_6_ complex: a KaiC hexamer (C_6_, PDB code: 3dvl)^16^, a ternary complex (_C_A_2_-B-_CI_C, PDB code: 5jwr)^26^ consisting of two C-terminal domains of KaiA dimer (_C_A_2_), a KaiB monomer (B) and a CI domain of KaiC (_CI_C), and a full-length structure of KaiA dimer (A_2_, PDB code: 1r8j)^11^. Here, we outline the initial modeling procedure of A_12_B_6_C_6_ (for details, see Supplementary note 2). First, we placed six _C_A_2_-B-c_I_C to C_6_ by superposing the _CI_C domain (green) (Supplementary Fig. 4a), thereby modeling the A_12_B_6_C_6_ complex, referred to as Complex 1. Note that Complex 1 does not have any _N_A domains. Complex 1 well agreed with the previously reported cryo-EM structure (RMSD = 3.9 Å between them). Next, we placed six full-length A_2_ to Complex 1 by superposing the _C1_A and _C2_A domains (Supplementary Fig. 4b). Note that the _N1_A (blue) and _N2_A (red) domains are derived from one A_2_ dimer and connected to the _C__1_A (cyan) and _C2_A(magenta) domains, respectively (inset of Supplementary Fig. 4b). Finally, we obtained an overall structure model of A_12_B_6_C_6_ complex, referred to as Complex 2. In this structure, each _N2_A domain (red) structurally overlapped with KaiB (yellow) (Supplementary Fig. 4c), indicating that the A_2_ dimer undergoes a conformational change in terms of the spatial arrangements of _N_A domains upon formation of the A_12_B_6_C_6_ complex.

#### Step 2: Generation of the structural candidates

To remove the KaiA-KaiB structural overlap in Complex 2, we systematically altered the positions and orientations of individual _N1_A and _N2_A domains belonging to one A_2_ dimer and gave the same conformation for the remaining five A_2_ dimer (applying the C6 symmetry around the first axis defined by B_6_C_6_). At this stage, we ignored the linkers connecting _N_A and _C_A domains to reduce the computational cost. As a result, we obtained ~20 million models of A_12_B_6_C_6_ complex as initial structural candidates (Supplementary Fig. 4d and see Supplementary note 4).

#### Step 3: Selection of models without linkers based on the SAXS and SANS data

We calculated the scattering curve for each of the models for screening based on the criterion of ${\chi }_{{{{{{\rm{SAXS}}}}}}}^{2} \, < \,10.0$ to the experimental SAXS data. We obtained about 400,000 models from the candidates generated in Step 2. The selected models were classified into three types, i.e., Types 1, 2, and 3, based on the location of the _N_A domains (Supplementary Fig. 5a). Type 1 holds both _N1_A (blue) and _N2_A (red) domains below the reference plane defined by the top plane of KaiB hexameric ring in the B_6_C_6_ subcomplex. Type 2 has one of _N_A domains below the reference plane while the other upper. In Type 3, both _N1_A and _N2_A domains are located upper the reference plane. The numbers of models were 331,000, 3000, and 62,000 for Types 1, 2, and 3, respectively.

Next, we evaluated the SAXS-selected models based on the iCM-SANS data as source information on the KaiA protomer conformations in the A_12_B_6_C_6_ complex. We found that only Type 1 gave the small χ^2^ values (${\chi }_{{{{{{\rm{SA}}}}}}{{{{{\rm{N}}}}}}{{{{{\rm{S}}}}}}}^{2} \, < \,3.0$) among the three types (Supplementary Fig. 5b), therefore leaving the Type 1 models as candidates. Thus, the combining of multiple experimental data can compensate their low resolution, underscoring the importance of multilateral evaluation in structural modeling of a huge complex.

#### Step 4: Linker formation

Through step 3, we selected 29,809 models with small χ^2^ values (${\chi }_{{{{{{\rm{SAXS}}}}}}}^{2} \, < \,6.0$ and ${\chi }_{{{{{{\rm{SANS}}}}}}}^{2} \, < \,1.5$) (structures within a white dotted box in Supplementary Fig. 5c) from the set of Type 1. Using the Rosetta program suite^51,52^, we attempted to complement these models with an _N_A–_C_A linker, which was ignored in the previous steps. Consequently, linker modeling was successful for about a quarter of the models (8608). For each of these models, about 100 multiple linker conformations were tested and the best model containing the linkers with the smallest χ^2^ value for the SAXS data was selected.

#### Step 5: Second selection of models with linkers by SAXS and SANS

We noticed that the linker addition affected their χ^2^ values for the SAXS and SANS (Supplementary Fig. 5d, e). We then re-evaluated and selected 1550 models for the overall A_12_B_6_C_6_ complex, which met the experimental SAXS and iCM-SANS profiles with ${\chi }_{{{{{{\rm{SAXS}}}}}}}^{2} \, < \,5.0$ and ${\chi }_{{{{{{\rm{SANS}}}}}}}^{2} \, < \,1.5$ (models within a white dotted square in Supplementary Fig. 5f).

**Fig. 3:**
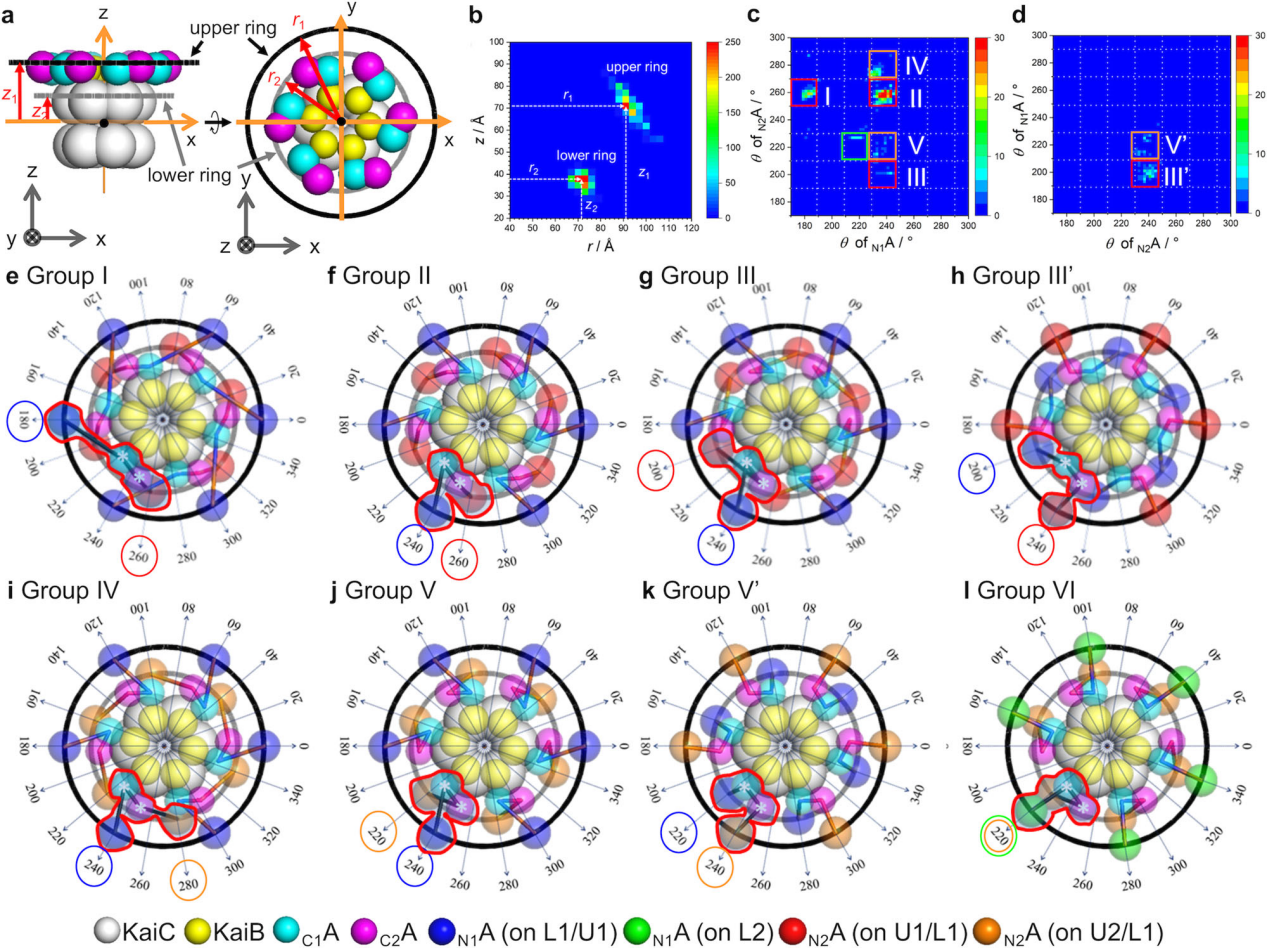
Classifications of structural models based on the location of _N_A domains. **a** Location of two rings on which _N_A domains distribute. Black and gray show upper and lower rings, respectively. The components are expressed with color spheres (see the indexes in the bottom). **b** Distribution of _N_A domains as a function of *r* and *z* directions. **c** Cell-correlation distribution map for the _N1_A and _N2_A domains, which locate on the upper and lower rings, respectively. Red, orange, and green squares show cell-combinations of U1-L1, U1-L2, and U2-L2 set, respectively (about the ‘cell’, see Supplementary Fig. 6a). Capital roman numerals, I–VI, indicate structural groups considering _N_A and _C_A domain connection. **d** Cell-correlation distribution map for the _N1_A and _N2_A domains, which locate on the lower and upper rings, respectively. Red and orange squares show cell-combinations of U1-L1 and U1-L2 set, respectively (about the ‘cell’, see Supplementary Fig. 6a). In Groups III’ and V’, the locational combination for the _N1_A and _N2_A domains are opposite to the cases of Groups III and V, respectively. **e**–**l** Top views of the eight structural groups. Radial arrows show azimuth angles on x-y plane. One A_2_ dimer including _C1_A (*θ* = 230°) and _C2_A (*θ* = 250°) domains (white asterisks) are surrounded by red lines. Blue and green circles indicate the cell positions of _N1_A domains, and red and orange circle also denote the cell positions of _N2_A domains. The components are also expressed with color spheres (see the indexes in the bottom).

Here we summarize the structural features of the selected models: although the _N_A domains occupy variable positions, we attempt to identify common features on positioning with respect to the B_6_C_6_ subcomplex. For this purpose, we defined the coordinate as shown in Fig. 3a and examined the positions of the _N_A domains. The space is divided into cells considering the symmetry of the cryo-EM structure, Complex 1 (for details of the space division and grouping procedure, see Supplementary note 5). The positions of _N_A domains were classified into two distinct groups: one distributed on the upper (U) rings surrounding Complex 1, and the other one, on the lower (L) ring (Fig. 3a, b). We also found significantly preferred positions for _N_A domains. In one A_2_ dimer, when one _N_A domain was located at the U ring, the other one was always at the lower L ring (Supplementary Fig. 6a). In each ring, two sets of possible positions were available for the _N_A domain, i.e. U1, U2, L1, and L2. In the U ring, the six _N_A domains distribute into mutually exclusive locations, U1 (blue spheres) and U2 (green spheres), each of which follows a six-fold symmetry (Supplementary Fig. 6a, b). In the L ring, the six _N_A domains also distribute into mutually exclusive locations with six-fold symmetry, L1 (red spheres) and L2 (orange spheres) (Supplementary Fig. 6c). In addition, we considered the linker connections between _N_A and _C_A domains in one A_2_ dimer, i.e., _N1_A–_C1_A and _N2_A–_C2_A (Fig. 3c, d). Taken together, the structural models were classified into eight groups as shown in Fig. 3e–l. In Groups I, II, and III, the _N1_A and _N2_A domains in one A_2_ dimer are located at U1 and L1, respectively. In Group III’, the positions of _N1_A and _N2_A domains are swapped from those in Group III, i.e., _N1_A and _N2_A are at L1 and U1, respectively. In Groups IV and V, the _N1_A and _N2_A domains in one A_2_ dimer are at U1 and L2, respectively. In Group V’, _N1_A, and _N2_A domains are reversely arranged as compared with those in Group V. In Group VI, _N1_A, and _N2_A domains are located at U2 and L2, respectively. The A_2_ dimer exhibits distinct conformations among the different groups as clarified in Fig. 3e–l (surrounded by red line).

In summary, in the L ring, the _N_A domains tend to be located at L1 or L2 (*θ* ~ 200 or 220 in Fig. 3 and Supplementary Fig. 6c) with six-fold symmetry. Their positions are between _C1_A and _C2_A domains belonging to two adjacent A_2_, respectively. At the U ring, the _N_A domains radially sit at the staggered positions with respect to its counterpart _N_A domains in the L ring. This exclusive rule is true for all the groups except Group VI. The structural features of the eight groups are summarized in Supplementary Table 3.

#### Step 6: Verification by MD simulation

**Fig. 4:**
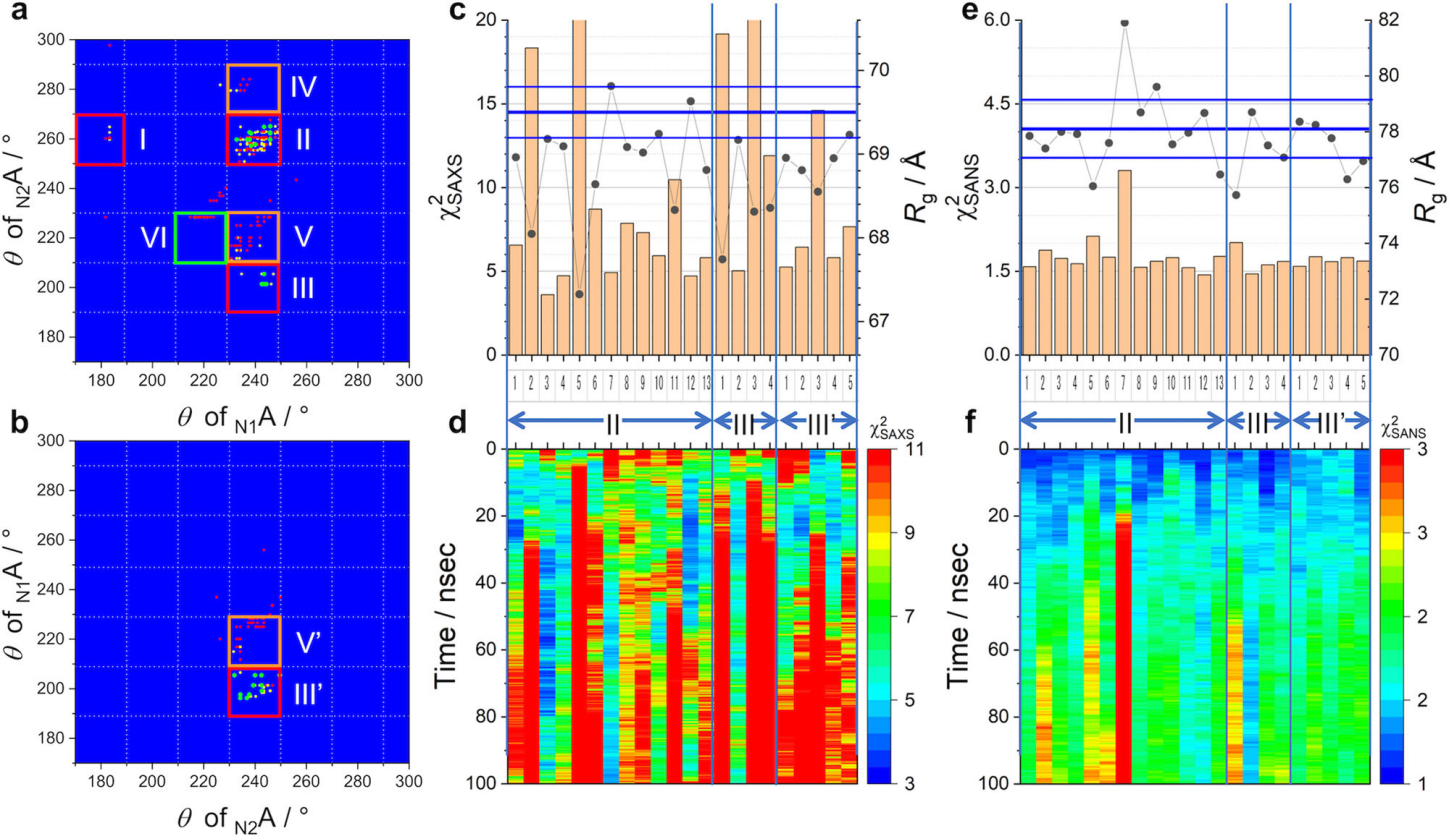
10 ns MD screening of selected models in eight groups and 100 ns MD verification of survived models (Model II-1 to II-13, III-1 to III-4, III’-1 to III’-5). **a**, **b** Distribution map of _N_A domains in the models after 10 ns MD simulation: **a** Groups I–VI and **b** Groups III’ and V’. Color denotes ${\chi }_{{{{{{\rm{SAXS}}}}}}}^{2}$ after 10 ns MD simulation. Green, yellow, and orange dots correspond to the structures with$\,{\chi }_{{{{{{\rm{SAXS}}}}}}}^{2} \, < \, 5.0$,$\,5.0\le {\chi }_{{{{{{\rm{SAXS}}}}}}}^{2} \, < \, 10.0$ and $10.0\le {\chi }_{{{{{{\rm{SAXS}}}}}}}^{2}$, respectively. **c**–**f** 100 ns MD simulation for 22 models. In each panel, numbers of horizontal axis indicate codes of models corresponding to Table 1. **c** ${\chi }_{{{{{{\rm{SAXS}}}}}}}^{2}$ (bars) and ${R}_{{{{{{\rm{g}}}}}},{{{{{\rm{SAXS}}}}}}}$ (black circles) for the SAXS profiles averaged over the simulation time (100 ns). **d** Time evolutions of ${\chi }_{{{{{{\rm{SAXS}}}}}}}^{2}$ in 100 ns MD simulations. **e** ${\chi }_{{{{{{\rm{SANS}}}}}}}^{2}$ (bars) and ${R}_{{{{{{\rm{g}}}}}},{{{{{\rm{SANS}}}}}}}$ (black circles) for the SANS profiles averaged over the simulation time (100 ns). **f** Time evolutions of ${\chi }_{{{{{{\rm{SANS}}}}}}}^{2}$ in 100 ns MD simulations. In panels **c** and **e**, thick blue lines show the experimental ${R}_{{{{{{\rm{g}}}}}}}\,$values and thin blue lines express the ranges of their errors.

In the screening procedure of Steps 1–5, the protein domains were treated as rigid bodies and only the exclusive volume of the molecules was considered. Thus, we further checked structural stability of the obtained models using MD simulation. As the first quick test for stability, we randomly selected the 384 models with$\,{\chi }_{{{{{{\rm{SAXS}}}}}}}^{2} < 5.0$, ${\chi }_{{{{{{\rm{SANS}}}}}}}^{2} < 1.25$ from the eight groups and performed 10-ns MD simulations. Figure 4a, b shows the relative locations of _N_A domains with ${\chi }_{{{{{{\rm{SAXS}}}}}}}^{2}$ after 10 ns. The values of ${\chi }_{{{{{{\rm{SAXS}}}}}}}^{2}$ are expressed in colors, as green, yellow, and red dots correspond to the structures with$\,{\chi }_{{{{{{\rm{SAXS}}}}}}}^{2} < 5.0$,$\,5.0\le {\chi }_{{{{{{\rm{SAXS}}}}}}}^{2} < 10.0$ and $10.0\le {\chi }_{{{{{{\rm{SAXS}}}}}}}^{2}$, respectively, and no model exceeds 1.5 of ${\chi }_{{{{{{\rm{SANS}}}}}}}^{2}$ after 10 ns MD simulation. The results clearly indicate that the models belonging to Groups II, III, and III’ maintained$\,{\chi }_{{{{{{\rm{SAXS}}}}}}}^{2} < 5.0$ but the model in the other groups yielded larger ${\chi }_{{{{{{\rm{SAXS}}}}}}}^{2}$. This suggests that the L1 position is more suitable for _N_A domains than L2. Moreover, Group I did not have any stable model, possibly because the stretching of the linker between _C1_A and _N1_A would make the structure unstable (Fig. 3e).

Considering the results above, we extended the simulation for 100 ns on 22 randomly selected models from Groups II, III’, and III that maintained stability during the 10 ns MD simulations. The number of selected models is 13, 4, and 5 for Groups II, III, and III’, respectively. For each model, we recorded trajectories every 20 ps and calculated the SAXS and SANS profiles of the 5000 snapshot structures. The averaged$\,{\chi }^{2}$ and ${R}_{{{\rm{g}}}}$ fits to the SAXS and SANS profiles are shown in Fig. 4c, e are summarized in Table 1. To find the structural models reproducing SAXS and SANS over the simulation time, we marked values with asterisks in Table 1, where two asterisks, one asterisk and no asterisk for$\,{\chi }^{2}$ denote ${\chi }^{2} > {\chi }_{0}^{2}\times 1.2$, ${\chi }_{0}^{2}\times 1.2\ge {\chi }^{2} > {\chi }_{0}^{2}$, and ${\chi }^{2}\le {\chi }_{0}^{2}$ (${\chi }_{0}^{2}$ = 5.0 for SAXS and ${\chi }_{0}^{2}$ = 1.5 for SANS), respectively, and they for ${R}_{{{\rm{g}}}}$ also denote $|\triangle {R}_{{{{{{\rm{g}}}}}}}| \, > \, 2\times {{{{{\rm{Error}}}}}}$, $2\times {{{{{\rm{Error}}}}}}\ge |\triangle {R}_{{{{{{\rm{g}}}}}}}| \, > \, {{{{{\rm{Error}}}}}}$, and $|\triangle {R}_{{{{{{\rm{g}}}}}}}|\le {{{{{\rm{Error}}}}}}$ ($\triangle {R}_{{{\rm{g}}}}={R}_{{{{{\rm{g}}}}},{{{{{\rm{MD}}}}}}}-{R}_{{{{{\rm{g}}}}},{{\mathrm exp}}}$), respectively. The structures that best reproduce the averaged scattering profiles are Model II-12 in Group II and Model III-2 in Group III. We further examined the time evolutions of ${\chi }_{{{{{{\rm{SAXS}}}}}}}^{2}$ and ${\chi }_{{{{{{\rm{SANS}}}}}}}^{2}$ as shown in Fig. 4d, f. Model II-12 shows that ${\chi }_{{{{{{\rm{SAXS}}}}}}}^{2}$ and ${\chi }_{{{{{{\rm{SANS}}}}}}}^{2}$ were initially small but gradually increased (after 50 ns). On the contrary, both ${\chi }_{{{{{{\rm{SAXS}}}}}}}^{2}$ and ${\chi }_{{{{{{\rm{SANS}}}}}}}^{2}$ of Model III-2 remained stable for all the 100 ns. In addition, trajectories and root mean square fluctuations (RMSFs) of center of mass (COM) of _C_A domains of Models II-12 and III-2 are calculated in the 100 ns MD simulations (Supplementary Fig. 7). In Model II-12, four _C2_A domains (C2-1, C2-3, C2-5, and C2-6) gives RMSFs of over 4.0 Å and the averaged value was also 4.0 Å. On the contrary, Model III-2 has only one _C2_A domain, which yielded a large fluctuation and then the averaged RMSF for the _C2_A domains was less than 4.0 Å (3.16 Å). This means if the fluctuation as seen in Model III-12 occurs, the structures of _C2_A domains would not be determined with a method like cryo-EM analysis. However, the cryo-EM study clearly observed the _C2_A domains in the A_12_B_6_C_6_ complex. Accordingly, we excluded Model II-12 from the structural candidate.

**Table 1:** Time averaged ${\chi }_{{{{{{\rm{SAXS}}}}}}}^{2}$, ${R}_{{{{{{\rm{g}}}}}},{{{{{\rm{SAXS}}}}}}}$, ${\chi }_{{{{{{\rm{SANS}}}}}}}^{2}$, and ${R}_{{{{{{\rm{g}}}}}},{{{{{\rm{SANS}}}}}}}$ of 100 ns MD simulated models.

Model	${\chi }_{{{{{{\rm{SAXS}}}}}}}^{2}$	${R}_{{{{{{\rm{g}}}}}},{{{{{\rm{SAXS}}}}}}}$	${\chi }_{{{{{{\rm{SANS}}}}}}}^{2}$	${R}_{{{{{{\rm{g}}}}}},{{{{{\rm{SANS}}}}}}}$
II-1	6.6**	69.0*	1.6*	77.8
II-2	18.3**	68.0**	1.9**	77.4
II-3	3.6	69.2	1.7*	78.0
II-4	4.7	69.1*	1.6*	77.9
II-5	41.1**	67.3**	2.1**	76.0**
I-6	8.7**	68.6**	1.7*	77.6
II-7	4.9	69.8	3.3**	81.9**
II-8	7.9**	69.1*	1.6	78.7
II-9	7.3**	69.0*	1.7*	79.6*
II-10	5.9**	69.2**	1.7*	77.6
II-11	10.5**	68.3**	1.6**	78.0
II-12	4.7	69.6	1.4	78.7
II-13	5.8*	68.8**	1.8**	76.5*
III-1	19.2**	67.7**	2.0**	75.7**
III-2	5.0	69.2	1.5	78.7
III-3	21.3**	68.3**	1.6*	77.5
III-4	11.9**	68.4**	1.7*	77.1
III’-1	5.2*	69.0*	1.6*	78.4
III’-2	6.4**	68.8**	1.8**	78.2
III’-3	14.6**	68.6**	1.7*	77.8
III’-4	5.8*	68.9*	1.7*	76.1**
III’-5	7.7**	69.2	1.7*	76.9*
Experiment		69.5 ± 0.3		78.1 ± 1.0

Two asterisks, one asterisk, and no asterisk for$\,{\chi }^{2}$ denote ${\chi }^{2} > {\chi }_{0}^{2}\times 1.2$, ${\chi }_{0}^{2}\times 1.2\ge {\chi }^{2} > {\chi }_{0}^{2}$, and ${\chi }^{2}\le {\chi }_{0}^{2}$ (${\chi }_{0}^{2}$ = 5.0 for SAXS and ${\chi }_{0}^{2}$ = 1.5 for SANS), respectively, and for ${R}_{{{\rm{g}}}}$ denote $\left|\triangle {R}_{{{{{{\rm{g}}}}}}}\right| > 2\times {{{{{\rm{Error}}}}}}$, $2\times {{{{{\rm{Error}}}}}}\ge \left|\triangle {R}_{{{{{{\rm{g}}}}}}}\right| > {{{{{\rm{Error}}}}}}$, and $\left|\triangle {R}_{{{{{{\rm{g}}}}}}}\right|\le {{{{{\rm{Error}}}}}}$ ($\triangle {R}_{{{\rm{g}}}}={R}_{{{\rm{g}}},{{\rm{MD}}}}-{R}_{{{\rm{g}}},{{\rm{exp}}}}$), respectively.

**Fig. 5:**
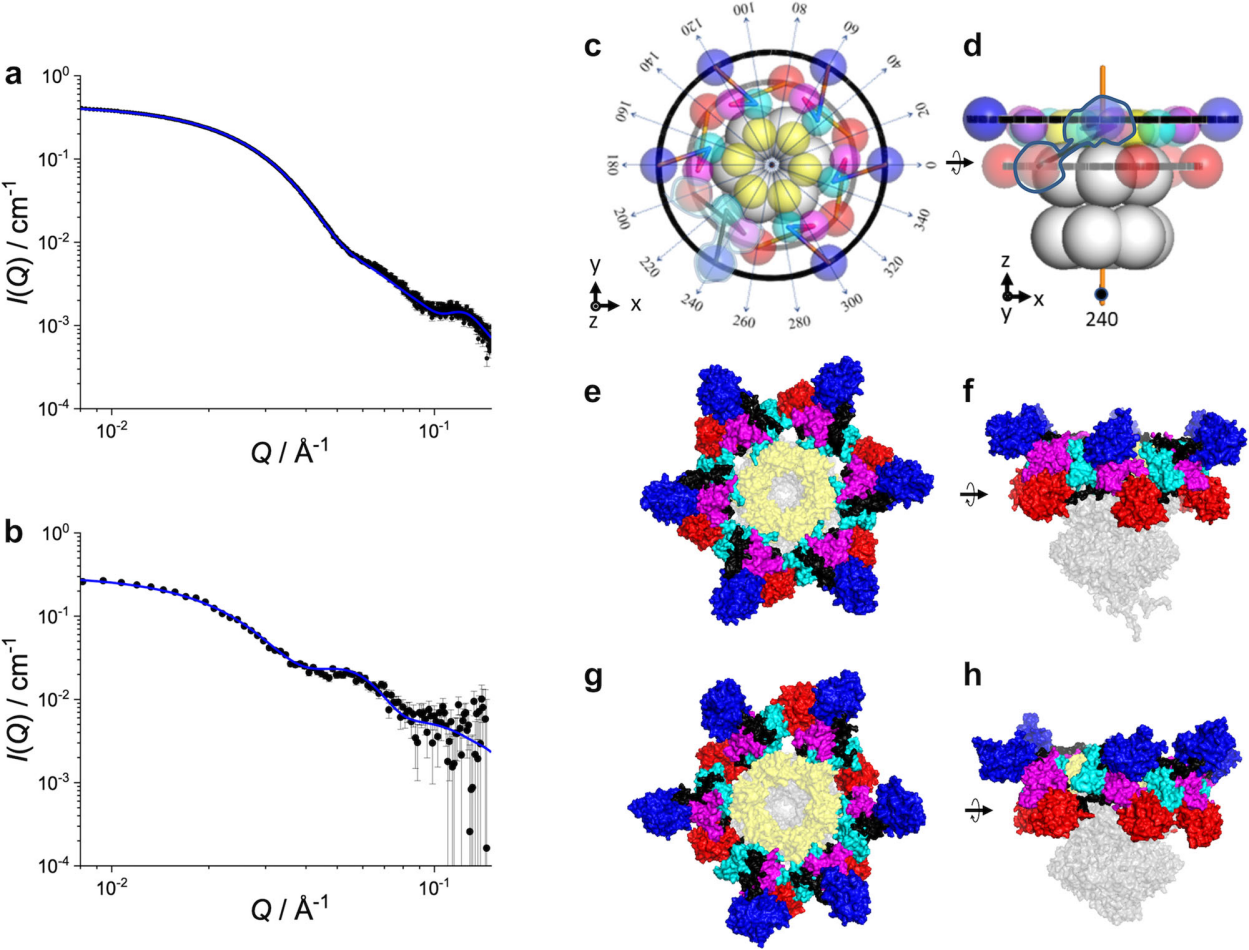
Best fit and stable structural model (Model III-2). **a** SAXS profiles. Black circles show the experimental profile and blue line expresses the SAXS profile averaged over the profiles calculated from 5000 MD-trajectories of Model III-2. **b** iCM-SANS profiles. Black circles show the experimental profile and blue line expresses the SANS profile averaged over the profiles calculated from 5000 MD-trajectories of Model III-2. **c** Top view and **d** side view of schematic structure of Model II-2. **e** Top view and **f** side view of initial structure, and **g** top view and **h** side view of the structure after 100 ns MD simulation. In panels **c**–**h**, the color codes for components are same as those in Fig. 3. Error bars in panels **a**, **b** represent standard deviation of the mean.

In conclusion, Model III-2 is considered as the best compromised model, reproducing satisfyingly both the SAXS (Fig. 5a) and SANS (Fig. 5b) data and remaining stable in 100 ns MD simulation. Figure 5c–h and Supplementary Movies 1 and 2 show its structure and dynamic fluctuations.

## Discussion

By integrating the experimental and computational approaches, the structure of the overall A_12_B_6_C_6_ complex was fully described. Although each _N_A domain could not be stabilized at a fixed position, we found their preferential positions in proposed models. Consequently, we successfully obtained a structural model, Model III-2, which does not only reproduce SEC-SAXS and SEC-iCM-SANS data but also remains structurally stable during the 100 ns-long MD simulation.

**Fig. 6:**
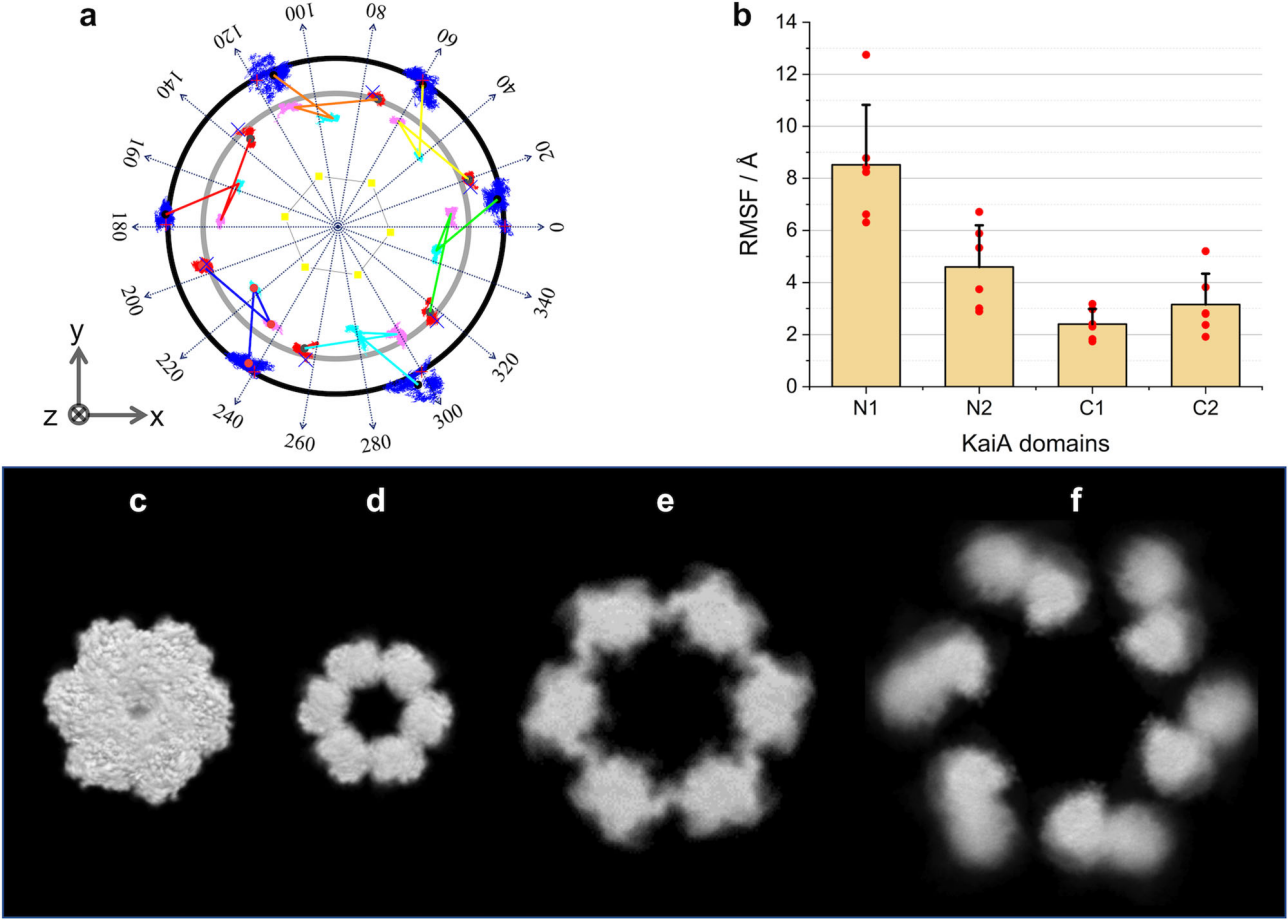
Dynamical fluctuations of domains in A_12_B_6_C_6_ complex (Model III-2). **a** Trajectories of the A_2_ domains. Blue, red, cyan magenta correspond to COMs of _N1_A, _N2_A, _C1_A, and _C2_A domains, respectively. Yellow dots express COMs of KaiB domains. Crosses show the initial positions in the 100 ns MD simulation. **b** The red points depict RMSFs of COMs of KaiA domains: Each _N1_A, _N2_A, _C1_A, and _C2_A has six domains. The thick orange bars show the averaged RMSFs with the error bars. Overlayed images in 100 ns MD simulations for **c** C_6_, **d** B_6_, **e** 12 _C_A domains, and **f** 12 _N_A domains.

Figure 6a shows the spatial fluctuations of KaiA domains of Model III-2 during the 100 ns simulation. Even though the COMs of _N_A domains are fluctuating, they remain around their initial positions. Furthermore, RMSF of COM of _N_A and _C_A domains (Fig. 6b) and overlayed respective images of C_6_ domain, B_6_ domain, _C_A domains and _N_A domains (Fig. 6c–f) clearly indicate that the _N_A domains are more mobile than the rest of the A_12_B_6_C_6_ complex. Also, the correlative motions between the _N_A domains of Model III-2 were examined during the 100 ns MD simulation. In the dynamical cross-correlation maps^53^ (Supplementary Fig. 8a, b), no clear correlation was observed among the six _N_A domains at the U ring, indicating that the motions of these domains are not synchronized. Concerning the _N_A domains at the L ring, only one pair (chains H and F) exhibit moderate correlation, basically indicating the non-synchronous motion of _N_A domains. All the results indicate the _N_A domains are randomly fluctuating, explaining why _N_A domains are invisible in the cryo-EM structure^25^.

It should be noted that the relative domain arrangement of the A_2_ in each of our A_12_B_6_C_6_ complex models (including Model III-2) is totally different from that in the crystal structure of A_2_ alone (PDB code: 1r8j) (Supplementary Fig. 8d, e) because they resolve structural overlap (pointed in Supplementary Fig. 4c). This suggests the possibility that the KaiA dimer structure in solution could undergo large conformational changes compared to that determined by the X-ray crystal analysis. To address this, we performed 500 ns MD simulations of the KaiA dimer in solution starting from the conformations in our A_12_B_6_C_6_ complex models (Mode lII-2) to examine the conformational stability of A_2_ (see Supplementary note 6). We computed SAXS profiles during the simulations to compare with the experimental one (described in AUC-SAXS of Materials and methods). Surprisingly, the ${\chi }^{2}$ values were kept relatively small and converged to ~3 after a few hundred nano-second, which were comparable to that for the crystal structure (${\chi }^{2}$=1.6) (Supplementary Fig. 8f, g). This suggests that the isolated KaiA dimer exhibits conformational variability in solution, including its crystallographic snapshots and simulated conformers in the A_12_B_6_C_6_ complex. These data suggest the A_2_ dimer potentially has multiple stable conformations and one of them could make induced fit when binding to the B_6_C_6_ complex.

**Fig. 7:**
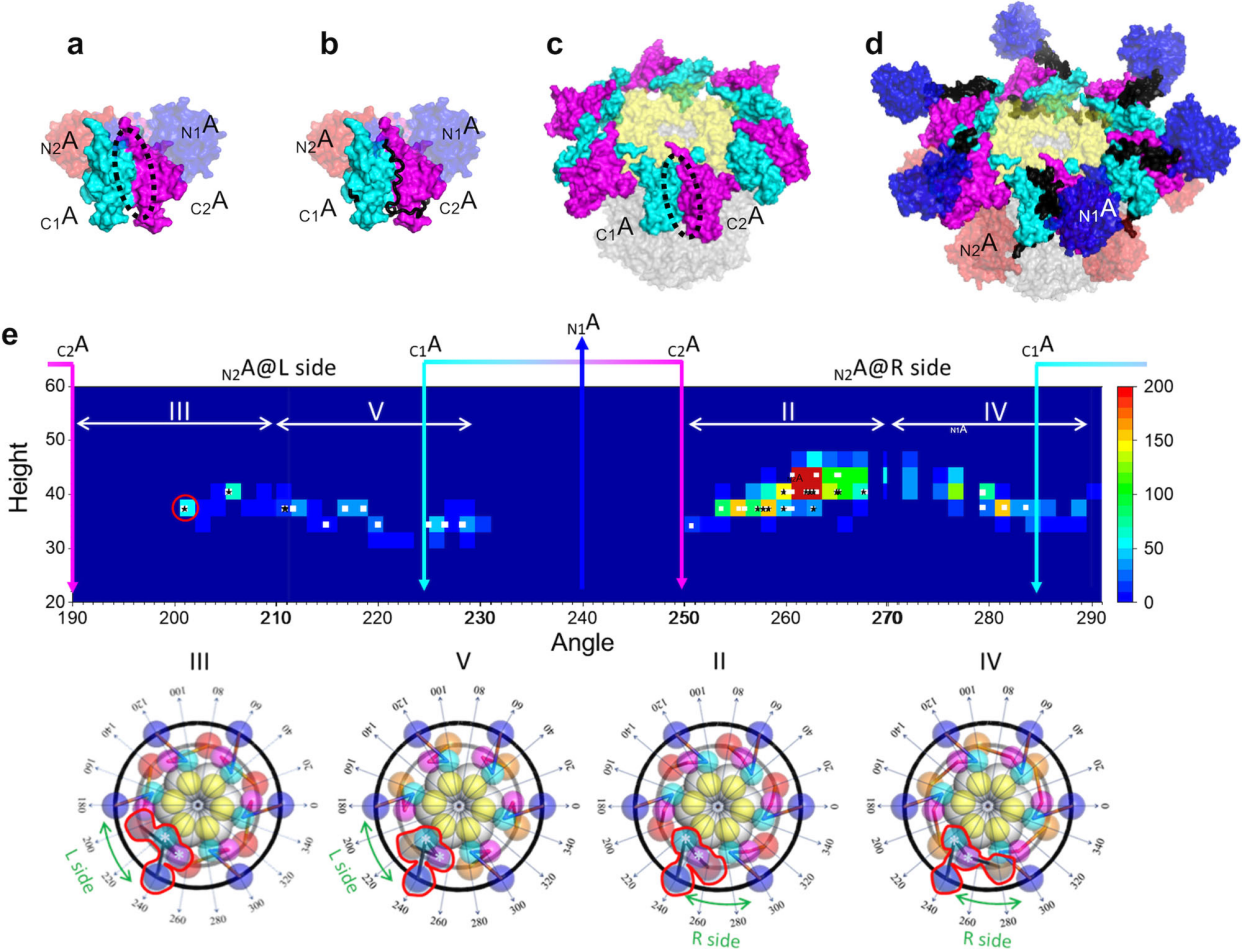
Positioning sites of _N_A domains of A_2_ protomers. **a** Hydrophobic surface (black dot circle) close to the dimeric interface of _C_A domains (cyan and magenta). **b** A complex of KaiA C-terminal domains (cyan and magenta) and disordered C-terminal segment of KaiC (black) (PDB code: 1suy). This is a part of A_2_C_6_ complex. **c** Complex 1 (same as the cryo-EM structure). The hydrophobic surfaces to be exposed to solvent. **d** Model III-2. The hydrophobic surfaces are masked by the _N1_A domains and the linkers. The coloring is as follows: _N1_A (blue), _N2_A (red), _C1_A (cyan), _C2_A (magenta), _N_A-_C_A linker (black), KaiB (yellow), and KaiC (gray). **e** (upper) Number distribution map of the _N2_A domain connecting the asterisked _C2_A domain (see lower inset) along the L ring of structure groups II–V: Horizontal and vertical axes are azimuth angle and height (*z*-axis) in the ABC complex coordinates (see the lower insets). The number of _N2_A domains is expressed of colors (see a scale bar). The white squares represent the models subjected to 10 ns MD simulation and the consequently verified models indicated by star marks. Red circle indicates the position of the _N2_A domain in Model III-2. Cyan, magenta, and blue arrows indicate the positioning angles of the _C1_A, _C2_A, and _N2_A domains, respectively. (Middle and Lower) The structural models of Groups II–IV as references. Colors of spheres for domains are the same as those in Fig. 3.

Next, we considered the role of _N_A domains related with their positions. During the circadian cycle, the KaiC phosphorylation switches the interaction modes with KaiA. Namely, the dephosphorylated C_6_ does not bind KaiB but interacts with A_2_ through its C-terminal tails, thereby forming the A_2_C_6_ complex (Process 1 in Supplementary Fig. 1)^16,54,55^. In contrast, the phosphorylated KaiC hexamer can form the B_6_C_6_ complex (Process 2 in Supplementary Fig. 1)^22,56,57^, which subsequently promotes direct binding of the KaiA dimer to the KaiB hexameric ring^8,26^, giving rise to the A_12_B_6_C_6_ complex (Process 3 in Supplementary Fig. 1)^25^, which has been characterized in this study. The KaiC dephosphorylation is accelerated in the ABC complex (Process 4 in Supplementary Fig. 1)^20,27,28^. In the A_2_C_6_ complex, a hydrophobic surface close to the dimeric interface of _C_A domains (a black dot circle in Fig. 7a) accommodates the C-terminal tail of KaiC (a black string in Fig. 7b). This binding surface appears to be exposed to solvent in the cryo-EM structure of A_12_B_6_C_6_ complex, which does not have electron densities of the _N_A domains (Fig. 7c). Intriguingly, this binding surface is masked by the _N1_A domain on the U ring in our structural model of the A_12_B_6_C_6_ complex (Model III-2), presumably hindering potential interaction via the KaiC C-terminal tail to form a larger complex (Fig. 7d). This is consistent with the AUC data indicating that the ABC complex did not form any complex larger than A_12_B_6_C_6_ complex (Supplementary Fig. 2b). Our findings provide a structural basis for the mechanism behind the precise circadian rhythm, that the formation of ABC complexes prevents the _N1_A domains on the U ring from additional interactions with any of the KaiC hexamers in the system, which would lead to infinite elongation of the complex.

During the 10 ns MD simulation, the _N2_A domains on the L ring traveled around the center between two adjacent _C_A domains (_C1_A and _C2_A domains, Fig. 7e). As each _C1_A domain bind one KaiB protomer in the B_6_C_6_ subcomplex, there exist six vacant spaces between adjacent _C_A domains (Fig. 3a). That is why the positioning of KaiA protomer in the complex appears unstable. However, the _N2_A domains of our models can be accommodated in these vacant spaces, which could stabilize the positioning of KaiA protomers.

In summary, we firstly succeeded in delineating the overall structure of A_12_B_6_C_6_ complex by the integration of experimental techniques such as SEC-SAXS, SEC-iCM-SANS, and AUC and computational modeling and simulations. The main issue to be resolved was to locate the _N_A domains missing in the cryo-EM structure. For this purpose, we used two different sets of scattering data, from SEC-SAXS reflecting the overall shape and from SEC-iCM-SANS extracting the conformational information of KaiA domains for screening the structures generated by the computational modeling. We emphasize that the SEC-iCM-SANS could reject the models, Types 2 and 3, which could not be excluded on the basis of the SAXS data only (Supplementary Fig. 5a). In addition, we demonstrated that MD simulation can be used for further model selection. In fact, the SAXS profiles of Model II-7 gave a small averaged ${\chi }_{{{{{{\rm{SAXS}}}}}}}^{2}$ value and they were stable during the 100 ns MD simulation (Fig. 4c, d). However, the SANS profiles clearly showed the difference among the models with small ${\chi }_{{{{{{\rm{SANS}}}}}}}^{2}$ (Fig. 4e, f). For example, the KaiA domains of Model II-7 was conformationally transformed from a six-fold symmetry to a pseudo-3-fold symmetry within a short time (20 ns). This latter conformation could well meet the experimental SAXS profiles but not the iCM-SANS profiles. Thus, this study demonstrates that the integrated approach of modern solution scattering methods, the SAXS and iCM-SANS techniques, and computational modeling and molecular dynamics simulation provide a powerful and generally applicable tool for resolving structures of supramolecular complexes harboring dynamically fluctuating domains/subunits like the KaiABC complex.

## Materials and methods

### Expression and purification of hydrogenated and deuterated Kai proteins

KaiA, KaiB, and KaiC from *Synechococcus sp*. PCC 7942 were expressed in *Escherichia coli*. KaiA was cloned into pET-28b according to the literature^60^. KaiA was expressed as hexahistidine (his)-tagged recombinant protein and purified after the cleavage of the his-tag as described previously^45^. KaiB was expressed as a glutathione S-transferase (GST)-tagged recombinant protein and purified after the cleavage of the GST-tag as described previously^61^. KaiC was expressed and purified as a Strep-tagged recombinant protein as described previously^9,21^. Here, we used the phosphorylation mimic KaiC in which one phosphorylation sites (S431) was substituted with an aspartate residue, because of its high affinity for KaiB.

For preparation of the deuterated proteins, the bacterial cells were grown in M9 minimal media containing glucose as a mixture with varying ratios of isotopically natural and fully deuterated glucose (1,2,3,4,5,6,6-D7, 98%, Cambridge Isotope Laboratories, Inc.), along with varying ratios of H_2_O and D_2_O as previously described^62^.

### Preparation for solution of A_12_B_6_C_6_ complex

We established a two-steps procedure to prepare a fully assembled ABC complex, which was expected to be A_12_B_6_C_6_ complex. In the first step, we produced B_6_C_6_ complex by mixing of KaiB and KaiC with the ration of 9:6 in the buffer, 20 mM Tris–HCl buffer (pH 8.0) containing 150 mM NaCl, 5 mM MgCl_2_, 0.5 mM EDTA, 1 mM DTT, and 1 mM ATP at 10 °C. Then, the BC complex was isolated from the mixture with SEC (Supplementary Fig. 2a). In the second step, oversaturating KaiA was added to the purified solution of B_6_C_6_ complex for preparing the A_12_B_6_C_6_ complex: The final mixing molar ratio was [KaiA]:[KaiB]:[KaiC] of 24:12:6 (Supplementary Fig. 2a). The formation of A_12_B_6_C_6_ complex was confirmed with AUC (Supplementary Fig. 2b). We used all hydrogenated Kai proteins in H_2_O buffer for the SAXS and AUC measurements. On the other hands, for preparing the samples of the SANS experiments, we used the proper combinations of hydrogenated and 75%-deuterated Kai proteins in D_2_O buffer: For example, to observe the conformations of KaiA domains in A_12_B_6_C_6_ complex, we used the combination of hydrogenated KaiA and 75%-deuterated forms of KaiB and KaiC.

### AUC

Sedimentation velocity-AUC (SV-AUC) measurements were conducted with a ProteomeLab XL-I (Beckman Coulter Inc., Brea, CA, USA). The optical path and the volume of the used cell were 12 mm and 400 μL, respectively. All measurements were performed using Rayleigh interference optics at 60,000 rpm at 30 °C. With this setting, we measured five samples (listed Supplementary Table 1), simple solutions of KaiA, KaiB, and KaiC, binary mixture solution of KaiB and KaiC, and ternary mixture solution of KaiA, KaiB, and KaiC. The first four sample were references and the last one was the sample solution including A_12_B_6_C_6_ complex as expected. The concentrations of the sample solutions of KaiA alone, KaiB alone, KaiC alone, KaiB-KaiC mixture, and KaiA-KaiB-KaiC mixture were 0.5, 0.5, 0.5, 0.6, and 1.0 mg/mL, respectively.

The AUC profile, the weight concentration distribution of particles in a solution *c*(*s*_20,w_), was obtained as a function of sedimentation coefficient by fitting the time evolution sedimentation data with Lamm formula using SEDFIT software (http://www.analyticalultracentrifugation.com/sedfit.htm)^63^. The sedimentation coefficient was normalized to be the value at 20 °C in pure water, *s*_20,w_. In addition, the molecular weight for each component was calculated using the corresponding peak value *s*_20,w_ and the friction ratio *f*/*f*_0_, which was also provided in the data reduction with SEDFIT (summarized in Supplementary Table 1).

For each component included in the ternary mixture sample, the weight fraction *r* was calculated from the corresponding peak-area in *c*(*s*_20,w_) and then the contribution ratio *t* in the forward scattering intensity of SAXS was also estimated from the molecular weight^50^. The results are listed in Supplementary Table 2.

### SEC-SAXS

SEC-SAXS experiment for the A_12_B_6_C_6_ complex was performed with Photon Factory BL-10C (Tsukuba, Japan) using UPLC ACQUITY (Waters Corp., Milford, MA, USA) integrated with a SAXS setup^47^. The wavelength of the injected X-ray and the detector were 1.50 Å and PILATUS3 2 M detector, respectively. The sample-to-detector distance were set to 3034.9 mm and then the covered *Q*-range was from 0.005 to 0.18 Å^−1^.

In the measurement, the sample solution of 250 μL of 11.7 mg/mL was loaded onto a Superose 6 increase 10/300GL (GE Healthcare, Chicago, IL, USA) pre-equilibrated with the buffer at a flow rate of 0.5 mL/min. During the elution of proteins, the flow rate was reduced to 0.05 mL/min. The SAXS and UV spectra at 280 nm were recoded every 20 and 10 s, respectively. The observed SAXS intensity was corrected for background, empty cell and buffer scatterings, and transmission factors and subsequently converted to the absolute scale using SAngler^47^. The unit of scattering intensity was converted to the absolute scale by referring to a standard scattering intensity of water at 293 K (1.632 × 10^−2^ cm^−1^)^64^.

There appeared a clear peak corresponding to A_12_B_6_C_6_ complex in the elusion chart (Supplementary Fig. 2c). We selected appropriate time ranges (indicated with the pink zones in Supplementary Fig. 2c) and made the average of the scattering intensities over the time range, where the averaged concentration was 1.02 mg/mL. We obtained the timing-coincident integrated scattering intensities in the *Q*-range from 0.008 to 0.015 Å^−1^. (Supplementary Fig. 2c).

### AUC-SAXS

AUC-SAXS experiment for A_2_ in solution was performed with ProteomeLab XL-I (Beckman Coulter Inc., Brea, CA, USA) and NANOPIX (RIGAKU Co., Ltd., Tokyo, Japan). SV-AUC measurement was carried out using Rayleigh interference optics at 60,000 rpm. The optical path and the volume of the AUC cell were 1.5 mm and 50 μL, respectively. SAXS measurement was conducted with the point-focused generator of a Cu-Kα source (wavelength = 1.54 Å) and HyPix-6000 detector. The sample-to-detector distance were set to 1280 mm and then the covered *Q*-range was from 0.01 to 0.2 Å^−1^. We subjected 1.0 mg/mL of KaiA solution to these measurements at 25 °C. AUC-SAXS treatment was conducted to eliminate the effect of aggregates and make the scattering data precise according to the previous report^50^.

### SEC-iCM-SANS

SEC-iCM-SANS experiments for hA_12_dB_6_dC_6_ and hA_12_hB_6_hC_6_ (as reference) were performed with the SEC system at D22 of the Institut Laue-Langevin (ILL), Grenoble, France. The neutron wavelength and the sample-to-detector distance were set to 6.0 Å and 5600 mm, respectively, and the covered *Q*-range was from 0.008 to 0.15 Å^−1^.

In the measurement, the sample solutions of 235 μL with 23.4 mg/mL (ternary mixture of hAdBdC) and 275 μL with 18.9 mg/mL (ternary mixture of hAhBhC) were loaded onto a Superose 6 Increase 10/300GL column (GE Healthcare, Chicago, IL, USA) with the D_2_O buffer at a flow rate of 0.5 mL/min. During the elution of proteins, the flow rate was reduced to 0.07 mL/min. The SANS data were collected for every 30 s and UV absorbance at 260 nm were recorded every 1 s. The observed SANS intensity was corrected for background, empty cell and buffer scatterings, and transmission factors and subsequently converted to the absolute scale using GRASP software using incident beam flux^65^. There appeared clear peaks corresponding to A_12_B_6_C_6_ complex in the elusion charts (Supplementary Fig. 2d, e). We selected the time range of FWHM of the peak and made the average of the scattering intensities over the selected time range (indicated with the pink zones in Supplementary Fig. 2d, e), where the averaged concentrations were 1.34 mg/mL and 1.27 mg/mL for the hA_12_hB_6_hC_6_ complex and hA_12_dB_6_dC_6_ complexes, respectively. We obtained the timing-coincident integrated scattering intensities in the *Q*-range from 0.008 to 0.015 Å^−1^. (Supplementary Fig. 2d, e).

### MD simulations for model verification

We performed MD simulations with the models that well reproduced the experimental SAXS and SANS profiles to examine whether they existed stably in solution. We performed conventional MD simulations (NVT) at a temperature of 300 K with no restraint using GROMACS^66–72^ with the Amber 14SB force field^73^ and the TIP3P water model^74^. The temperature was controlled using the V-rescale method^75^. The Na^+^ and Cl^−^ ions were added to neutralize the system and maintain the salt concentration at 150 mM. The salt concentration was set at the same value as the scattering experiment (see Supplementary Table 4 for other details).

SAXS and iCM-SANS profiles of MD snapshot structures were calculated with CRYSOL^76^ and CRYSON^77^. The smearing effect of instrumental resolution on iCM-SANS profile was considered with the resolution provided by GRASP^65^.

### Dynamical cross-correlation map

The dynamical cross-correlation^53^ is calculated for the C${{{{{\rm{\alpha }}}}}}$ atom pairs in the _N_A domains during the 100 ns-long MD simulation. The correlation coefficient between *i*th and *j*th C${{{{{\rm{\alpha }}}}}}$ atoms, whose positions are ${{{{{{\bf{r}}}}}}}_{{{{{{\rm{i}}}}}}}\,$and ${{{{{{\bf{r}}}}}}}_{j}$ respectively, is defined as ${c}_{{ij}}/({c}_{{ii}}^{1/2}{c}_{{jj}}^{1/2})$, where ${c}_{{ij}}=\langle ({{{{{{\bf{r}}}}}}}_{i}-\langle {{{{{{\bf{r}}}}}}}_{i}\rangle )\cdot ({{{{{{\bf{r}}}}}}}_{j}-\langle {{{{{{\bf{r}}}}}}}_{j}\rangle )\rangle$ and $\langle \rangle$ denotes the average during the simulation. Before the calculation, the atoms in each KaiA dimer are structurally aligned by the RMS-fitting so that all the _C_A_2_ domains during the simulation overlap each other. In this way, the comparison of the motions of the six KaiA dimers is straightforward. The correlations between upper _N_A domains and those between lower _N_A domains are computed (Supplementary Fig. 8a, b).

### Statistics and reproducibility

The fittings for Guinier formula to derive *I*(0) and *R*_g_ were performed with the linear least-square method. The errors were defined as the standard deviation.

### Reporting summary

Further information on research design is available in the Nature Research Reporting Summary linked to this article.

## Supplementary information

Transparent Peer Review File

Supplementary Information File

Description of Additional Supplementary Files

Supplementary Movie 1

Supplementary Movie 2

Supplementary Data 1

Supplementary Data 2

Reporting Summary

## Data Availability

The datasets generated and analyzed during the current study are available from the corresponding authors on reasonable request. The SEC-SAXS and SEC-iCM-SANS data are deposited in SASBDB under SASDNJ2 and SASDNK2, respectively^58^. The 100 ns MD trajectory of the representative model of the A_12_B_6_C_6_ complex (III-2) are deposited in to the Biological Structure Model Archive (BSM-Arc) under BSM-ID BSM00030^59^.

## References

[1] Bonomi M, Vendruscolo M (2019). Determination of protein structural ensembles using cryo-electron microscopy. Curr. Opin. Struct. Biol..

[2] Patil NK, Bohannon JK, Hernandez A, Patil TK, Sherwood ER (2019). Regulation of leukocyte function by citric acid cycle intermediates. J. Leukoc. Biol..

[3] Sora V (2020). Structure and dynamics in the ATG8 family from experimental to computational techniques. Front. Cell Dev. Biol..

[4] Ziegler SJ, Mallinson SJ, John PCS, Bomble YJ (2020). Advances in integrative structural biology: Towards understanding protein complexes in their cellular context. Comput. Struct. Biotechnol. J..

[5] Yamaguchi HQ, Ode KL, Ueda HR (2021). A design principle for posttranslational chaotic oscillators. iScience.

[6] Ishiura M (1998). Expression of a gene cluster *kaiABC* as a circadian feedback process in cyanobacteria. Science.

[7] Nakajima M (2005). Reconstitution of circadian oscillation of cyanobacterial KaiC phosphorylation in vitro. Science.

[8] Chang YG (2015). Circadian rhythms. A protein fold switch joins the circadian oscillator to clock output in cyanobacteria. Science.

[9] Oyama K, Azai C, Nakamura K, Tanaka S, Terauchi K (2016). Conversion between two conformational states of KaiC is induced by ATP hydrolysis as a trigger for cyanobacterial circadian oscillation. Sci. Rep..

[10] Vakonakis I (2004). NMR structure of the KaiC-interacting C-terminal domain of KaiA, a circadian clock protein: implications for KaiA-KaiC interaction. Proc. Natl Acad. Sci. USA.

[11] Ye S, Vakonakis I, Ioerger TR, LiWang AC, Sacchettini JC (2004). Crystal structure of circadian clock protein KaiA from *Synechococcus elongatus*. J. Biol. Chem..

[12] Hitomi K, Oyama T, Han S, Arvai AS, Getzoff ED (2005). Tetrameric architecture of the circadian clock protein KaiB. A novel interface for intermolecular interactions and its impact on the circadian rhythm. J. Biol. Chem..

[13] Iwase R (2005). Functionally important substructures of circadian clock protein KaiB in a unique tetramer complex. J. Biol. Chem..

[14] Mori T (2002). Circadian clock protein KaiC forms ATP-dependent hexameric rings and binds DNA. Proc. Natl Acad. Sci. USA.

[15] Hayashi F (2003). ATP-induced hexameric ring structure of the cyanobacterial circadian clock protein KaiC. Genes Cells.

[16] Pattanayek R (2004). Visualizing a circadian clock protein: crystal structure of KaiC and functional insights. Mol. Cell.

[17] Yunoki, Y. et al. ATP hydrolysis by KaiC promotes its KaiA binding in the cyanobacterial circadian clock system. *Life Sci. Alliance*10.26508/lsa.201900368 (2019). 10.26508/lsa.201900368 PMC6549140 31160381

[18] Pattanayek R (2006). Analysis of KaiA-KaiC protein interactions in the cyano-bacterial circadian clock using hybrid structural methods. EMBO J..

[19] Pattanayek R (2011). Combined SAXS/EM based models of the *S. elongatus* post-translational circadian oscillator and its interactions with the output His-kinase SasA. PLoS ONE.

[20] Phong C, Markson JS, Wilhoite CM, Rust MJ (2013). Robust and tunable circadian rhythms from differentially sensitive catalytic domains. Proc. Natl Acad. Sci. USA.

[21] Sugiyama M (2016). Structural characterization of the circadian clock protein complex composed of KaiB and KaiC by inverse contrast-matching small-angle neutron scattering. Sci. Rep..

[22] Kageyama H (2006). Cyanobacterial circadian pacemaker: Kai protein complex dynamics in the KaiC phosphorylation cycle in vitro. Mol. Cell.

[23] Mori T (2007). Elucidating the ticking of an in vitro circadian clockwork. PLoS Biol..

[24] Akiyama S, Nohara A, Ito K, Maeda Y (2008). Assembly and disassembly dynamics of the cyanobacterial periodosome. Mol. Cell.

[25] Snijder J (2017). Structures of the cyanobacterial circadian oscillator frozen in a fully assembled state. Science.

[26] Tseng R (2017). Structural basis of the day-night transition in a bacterial circadian clock. Science.

[27] Brettschneider C (2010). A sequestration feedback determines dynamics and temperature entrainment of the KaiABC circadian clock. Mol. Syst. Biol..

[28] Mori T (2018). Revealing circadian mechanisms of integration and resilience by visualizing clock proteins working in real time. Nat. Commun..

[29] Nishimura H (2002). Mutations in KaiA, a clock protein, extend the period of circadian rhythm in the cyanobacterium Synechococcus elongatus PCC 7942. Microbiology (Reading).

[30] Chen, Q., Liu, S., Yang, L., Zhang, L. & Li, J. The reversible function switching of the circadian clock protein KaiA is encoded in its structure. *Biochim. Biophys. Acta* **1861**, 2535–2542, (2017). 10.1016/j.bbagen.2017.08.012 28844977

[31] Fejgin, L. A. & Svergun, D. I. *Structure Analysis by Small-angle X-ray and Neutron Scattering*. (Plenum Press, 1987).

[32] Svergun DI (1999). Restoring low resolution structure of biological macromolecules from solution scattering using simulated annealing. Biophys. J..

[33] Bernadó P, Shimizu N, Zaccai G, Kamikubo H, Sugiyama M (2018). Solution scattering approaches to dynamical ordering in biomolecular systems. Biochim. Biophys. Acta.

[34] Bonomi M, Heller GT, Camilloni C, Vendruscolo M (2017). Principles of protein structural ensemble determination. Curr. Opin. Struct. Biol..

[35] Rout MP, Sali A (2019). Principles for integrative structural biology studies. Cell.

[36] Lattman, E. E., Grant, T. D. & Snell, E. H. in *Biological Small Angle Scattering: Theory and Practice*. Vol. 29 (Oxford University Press, 2018).

[37] David G, Pérez J (2009). Combined sampler robot and high-performance liquid chromatography: a fully automated system for biological small-angle X-ray scattering experiments at the Synchrotron SOLEIL SWING beamline. J. Appl. Crystallogr..

[38] Ryan TM (2018). An optimized SEC-SAXS system enabling high X-ray dose for rapid SAXS assessment with correlated UV measurements for biomolecular structure analysis. J. Appl. Crystallogr..

[39] Inoue R (2019). Newly developed laboratory-based size exclusion chromatography small-angle x-ray scattering system (La-SSS). Sci. Rep..

[40] Paissoni C, Jussupow A, Camilloni C (2019). Martini bead form factors for nucleic acids and their application in the refinement of protein–nucleic acid complexes against SAXS data. J. Appl. Crystallogr..

[41] Jussupow A (2020). The dynamics of linear polyubiquitin. Sci. Adv..

[42] Okuda A (2021). Solution structure of multi-domain protein ER-60 studied by aggregation-free SAXS and coarse-grained-MD simulation. Sci. Rep..

[43] Sugiyama M (2014). Conformational characterization of a protein complex involving intrinsically disordered protein by small-angle neutron scattering using the inverse contrast matching method: a case study of interaction between α-synuclein and PbaB tetramer as a model chaperone. J. Appl. Crystallogr..

[44] Yogo R (2017). Characterization of conformational deformation-coupled interaction between immunoglobulin G1 Fc glycoprotein and a low-affinity Fcγ receptor by deuteration-assisted small-angle neutron scattering. Biochem. Biophys. Rep..

[45] Sekiguchi T (2019). Mutational and combinatorial control of self-assembling and disassembling of human proteasome α subunits. Int. J. Mol. Sci..

[46] Inoue R (2021). Elucidation of the mechanism of subunit exchange in αB crystallin oligomers. Sci. Rep..

[47] Shimizu, N. et al. in *AIP Conference Proceedings*. 050017 (AIP Publishing LLC, 2016).

[48] Johansen NT, Pedersen MC, Porcar L, Martel A, Arleth L (2018). Introducing SEC–SANS for studies of complex self-organized biological systems. Acta Crystallogr. Sect. D Struct. Biol..

[49] Sato N (2021). A feasibility study of inverse contrast-matching small-angle neutron scattering method combined with size exclusion chromatography using antibody interactions as model systems. J. Biochem..

[50] Morishima K (2020). Integral approach to biomacromolecular structure by analytical-ultracentrifugation and small-angle scattering. Commun. Biol..

[51] Mandell DJ, Coutsias EA, Kortemme T (2009). Sub-angstrom accuracy in protein loop reconstruction by robotics-inspired conformational sampling. Nat. Methods.

[52] Huang P-S (2011). RosettaRemodel: a generalized framework for flexible backbone protein design. PLoS ONE.

[53] Hünenberger PH, Mark AE, van Gunsteren WF (1995). Fluctuation and cross-correlation analysis of protein motions observed in nanosecond molecular dynamics simulations. J. Mol. Biol..

[54] Iwasaki H, Nishiwaki T, Kitayama Y, Nakajima M, Kondo T (2002). KaiA-stimulated KaiC phosphorylation in circadian timing loops in cyanobacteria. Proc. Natl Acad. Sci. USA.

[55] Kim YI, Dong G, Carruthers CW, Golden SS, LiWang A (2008). The day/night switch in KaiC, a central oscillator component of the circadian clock of cyanobacteria. Proc. Natl Acad. Sci. USA.

[56] Kitayama Y, Iwasaki H, Nishiwaki T, Kondo T (2003). KaiB functions as an attenuator of KaiC phosphorylation in the cyanobacterial circadian clock system. EMBO J..

[57] Nishiwaki T (2007). A sequential program of dual phosphorylation of KaiC as a basis for circadian rhythm in cyanobacteria. EMBO J..

[58] Valentini, E., Kikhney, A. G., Previtali, G., Jeffries, C. M. & Svergun, D. I. SASBDB, a repository for biological small-angle scattering data. *Nucleic Acids Res*. **43**, D357–D363 (2015). 10.1093/nar/gku1047 PMC4383894 25352555

[59] Bekker, G.-J., Kawabata, T. & Kurisu, G. The Biological Structure Model Archive (BSM-Arc): an archive for in silico models and simulations. *Biophys. Rev.* **12**, 371–375 (2020). 10.1007/s12551-020-00632-5 PMC7242595 32026396

[60] Chang YG, Kuo NW, Tseng R, LiWang A (2011). Flexibility of the C-terminal, or CII, ring of KaiC governs the rhythm of the circadian clock of cyanobacteria. Proc. Natl Acad. Sci. USA.

[61] Murakami, R. et al. Cooperative binding of KaiB to the KaiC hexamer ensures accurate circadian clock oscillation in cyanobacteria. *Int. J. Mol. Sci*. 10.3390/ijms20184550 (2019). 10.3390/ijms20184550 PMC6769508 31540310

[62] Okuda A (2021). Deuteration aiming for neutron scattering. Biophys. Physicobiol..

[63] Schuck P (2000). Size-distribution analysis of macromolecules by sedimentation velocity ultracentrifugation and lamm equation modeling. Biophys. J..

[64] Orthaber D, Bergmann A, Glatter O (2000). SAXS experiments on absolute scale with Kratky systems using water as a secondary standard. J. Appl. Crystallogr..

[65] Dewhurst, C. *GRASP*. https://www.ill.eu/users/support-labs-infrastructure/software-scientific-tools/grasp. (Institut Laue-Langevin, 2020).

[66] Berendsen HJ, van der Spoel D, van Drunen R (1995). GROMACS: a message-passing parallel molecular dynamics implementation. Computer Phys. Commun..

[67] Lindahl E, Hess B, Van Der Spoel D (2001). GROMACS 3.0: a package for molecular simulation and trajectory analysis. Mol. Model. Annu..

[68] Van Der Spoel D (2005). GROMACS: fast, flexible, and free. J. Comput. Chem..

[69] Hess B, Kutzner C, Van Der Spoel D, Lindahl E (2008). GROMACS 4: algorithms for highly efficient, load-balanced, and scalable molecular simulation. J. Chem. Theory Comput..

[70] Pronk S (2013). GROMACS 4.5: a high-throughput and highly parallel open source molecular simulation toolkit. Bioinformatics.

[71] Páll, S., Abraham, M. J., Kutzner, C., Hess, B. & Lindahl, E. Tackling exascale software challenges in molecular dynamics simulations with GROMACS. In *Solving software challenges for exascale*, (eds Markidis, S. & Laure, E.), 3–27, (Springer, 2015).

[72] Abraham MJ (2015). GROMACS: High performance molecular simulations through multi-level parallelism from laptops to supercomputers. SoftwareX.

[73] Maier JA (2015). ff14SB: improving the accuracy of protein side chain and backbone parameters from ff99SB. J. Chem. Theory Comput..

[74] Jorgensen WL, Chandrasekhar J, Madura JD, Impey RW, Klein ML (1983). Comparison of simple potential functions for simulating liquid water. J. Chem. Phys..

[75] Bussi G, Donadio D, Parrinello M (2007). Canonical sampling through velocity rescaling. J. Chem. Phys..

[76] Franke D (2017). ATSAS 2.8: a comprehensive data analysis suite for small-angle scattering from macromolecular solutions. J. Appl. Crystallogr..

[77] Svergun D (1998). Protein hydration in solution: experimental observation by x-ray and neutron scattering. Proc. Natl Acad. Sci. USA.

