# Supplementary material for: Overall structure of fully assembled cyanobacterial KaiABC circadian clock complex by an integrated experimental-computational approach
Source: Commun Biol. 2022 Mar 10;5:184. doi: 10.1038/s42003-022-03143-z (PMC8913699; doi:10.1038/s42003-022-03143-z)
Supplement: Supplementary file 2 — Supplementary Information File [file 42003_2022_3143_MOESM2_ESM.pdf]

Supplementary information

Overall structure of fully assembled cyanobacterial KaiABC circadian clock complex by an integrated experimental-computational approach

Yasuhiro Yunoki[1, 2, #, ǂ], Atsushi Matsumoto[3, ǂ], Ken Morishima[4], Anne Martel[5], Lionel Porcar[5], Nobuhiro Sato[4], Rina Yogo[1, 2, †], Taiki Tominaga[6], Rintaro Inoue[4], Maho Yagi-Utsumi[1, 2], Aya Okuda[4], Masahiro Shimizu[4], Reiko Urade[4], Kazuki Terauchi[7], Hidetoshi Kono[3, *], Hirokazu Yagi[2, *], Koichi Kato[1, 2, *] and Masaaki Sugiyama[4, *]

[1] Exploratory Research Center on Life and Living Systems (ExCELLS) and Institute for Molecular Science (IMS), National Institutes of Natural Sciences, 5-1 Higashiyama, Myodaiji-cho, Okazaki, 444-8787, Japan.

[2] Graduate School of Pharmaceutical Sciences, Nagoya City University, 3-1 Tanabe-dori, Mizuhoku, Nagoya 467-8603, Japan.

[3] National Institutes for Quantum and Radiological Science and Technology (QST), Umemidai, Kizu, Kyoto 619-0215, Japan.

[4] Institute for Integrated Radiation and Nuclear Science, Kyoto University, 2-1010 Asashironishi, Kumatori, Sennan-gun, Osaka, 590-0494, Japan.

[5] Institut Laue-Langevin, 6 Rue Jules Horowitz, Grenoble 38042, France.

[6] Neutron Science and Technology Center, Comprehensive Research Organization for Science and Society (CROSS), Tokai, Ibaraki 319-1106, Japan

[7] Graduate School of Life Sciences, Ritsumeikan University, 1-1-1 Noji-higashi, Kusatsu, Shiga 525-8577, Japan

ǂ equal contributions

Present addresses:

[#]Institute for Integrated Radiation and Nuclear Science, Kyoto University, 2-1010 Asashironishi, Kumatori, Sennan-gun, Osaka, 590-0494, Japan.

[†]Biomedical Research Centre, School of Biomedical Engineering, The University of British Columbia, 2222 Health Sciences Mall, Vancouver, BC Canada V6T 1Z3

[*]To whom correspondence should be addressed. E-mail: sugiyama.masaaki.5n@kyoto-u.ac.jp, hyagi@phar.nagoya-cu.ac.jp, kkatonmr@ims.ac.jp, kono.hidetoshi@qst.go.jp

Supplementary Figures

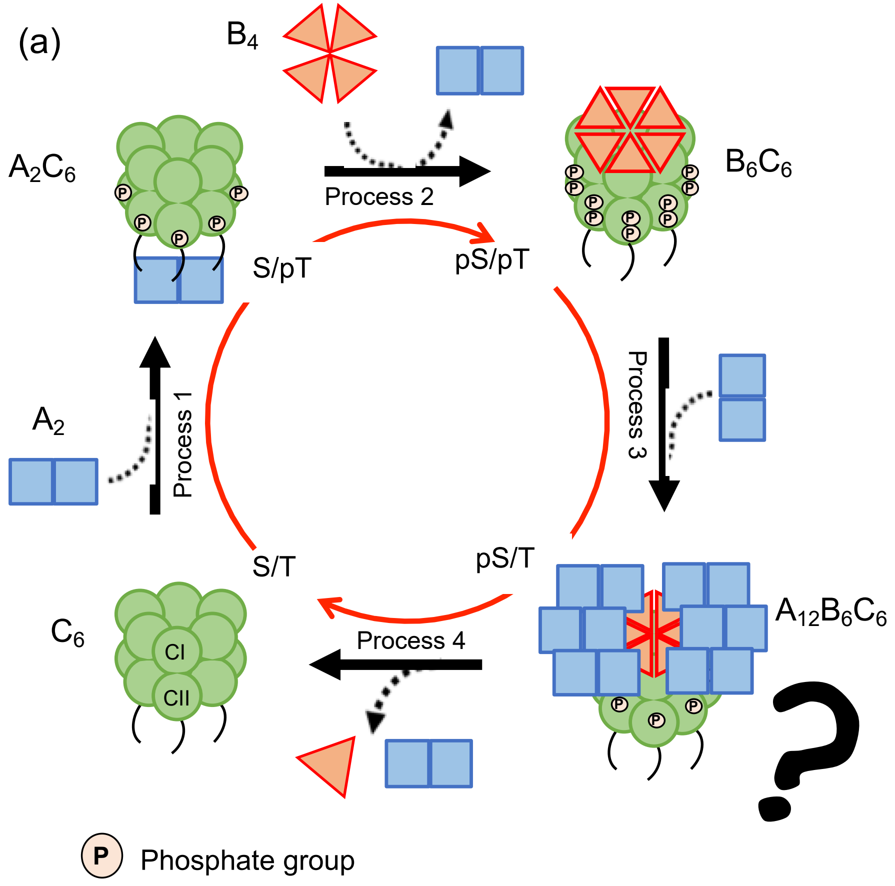

(b)

KaiA
GSHMLSQIAICIWVESTAILQDCQRALSADRYQLQVCESGEMLLEYAQT
HRDQIDCLILVAANPSFRAVVQQLCFEGVVVPAIVVGDRDSEDPDEPAK
EQLYHSAELHLGIHQLEQLPYQVDAALAEFLRLAPVETMADHIMLMGAN
HDPELSSQQRDLAQRLQERLGYLGVYYKRDPDRFLRNLPAYESQKLH
QAMQTSYREIVLSYFSPNSNLNQSIDNFVNMAFFADVPVTKVVEIHMEL
MDEFAKKLRVEGRSEDILLDYRLTLIDVIAHLCEMYRRSIPRET

KaiB
GPLGSMSPRKTYILKLYVAGNTPNSVRALKTLKNILEVEFQGVYALKVID
VLKNPQLAEEDKILATPTLAKVLPLPVRRIIGDLSDREKVLIGLDLLYGEL
QDSDDF

KaiC_S431D
MASWSHPQFEKGATSAEMTSPNNNSEHQAIAKMRTMIEGFDDISHGG
LPIGRSTLVSGTSGTGKTLFSIQFLYNGIIEFDEPGVFVTFEETPQDIIKNA
RSFGWDLAKLVDEGKLFILDASPDPEGQEVVGGFDLSALIERINYAIQKY
RARRVSIDSVTSVFQQYDASSVVRRELFRLVARLKQIGATTVMTTERIEE
YGPIARYGVEEFVSDNVVILRNVLEGERRRRTLEILKLRGTSHMKGEYP
FTITDHGINIFPLGAMRLTQRSSNVRVSSGVVRLDEMCGGGFFKDSIILA
TGATGTGKTLLVSRFVENACANKERAILFAYEESRAQLLRNAYSWGMD
FEEMERQNLLKIVCAYPESAGLEDHLQIIKSEINDFKPARIAIDSLSALAR
GVSNNAFRQFVIGVTGYAKQEEITGLFTNTSDQFMGAHSITDSHIDTITD
TIILLQYVEIRGEMSRAINVFKMRGSWHDKAIREFMISDKGPDIKDSFRN
FERIISGSPTRITVDEKSELSRIVRGVQEKGPES

Supplementary Figure 1. A proposed oscillation scheme in cyanobacterial circadian clock system (Kai clock system). (a) Blue squares, orange triangles and green spheres show monomers of KaiA, KaiB and KaiC, respectively. There are two phosphorylated cites, i.e. Ser431 and Thr432, in a CII domain of KaiC. The phosphorylation cycle is as follows: ST→pST→pSpT→pST→ST (p indicates a phosphorylated residue.) The

phosphorylation-dephosphorylation cycle of KaiC is coupled with its association and dissociation with KaiA and KaiB. Process 1: The formation of A_2C_6 complex and KaiC phosphorylation of ST→pST. Process 2: The formation of B_6C_6 complex and KaiC phosphorylation of pST→pSpT. Process 3: The formation of ABC complex and KaiC dephosphorylation of pSpT→pST. Process 4: The deformation of ABC complex and KaiC dephosphorylation of pST→ST. (b) Clock protein constructs from *Synechococcus sp.* PCC 7942 used in this study.

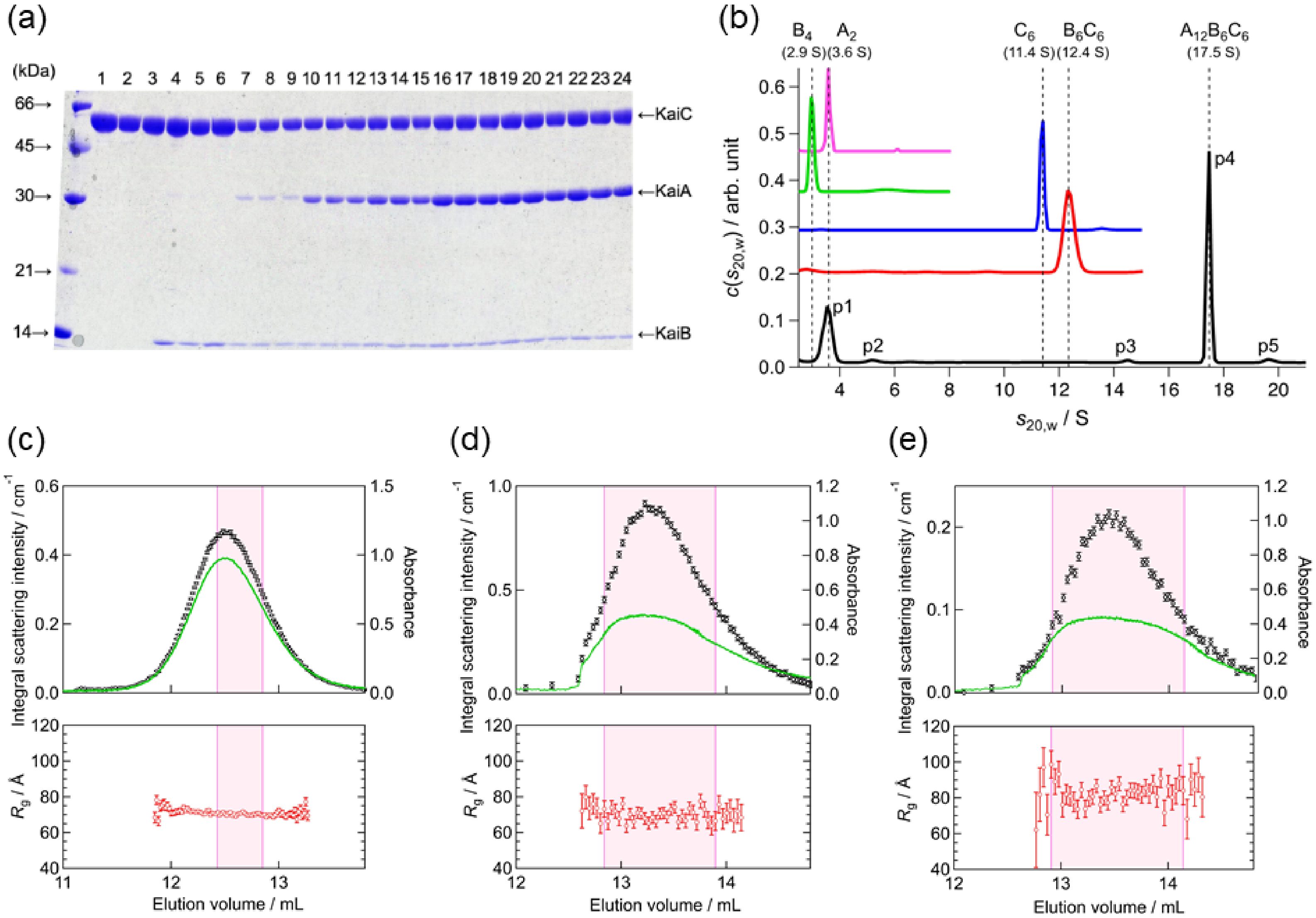

(a)
(kDa)
66
45
30
21
14
1 2 3 4 5 6 7 8 9 10 11 12 13 14 15 16 17 18 19 20 21 22 23 24
KaiC
KaiA
KaiB
(b)
B4 (2.9 S)
A2 (3.6 S)
C6 (11.4 S)
B6C6 (12.4 S)
A12B6C6 (17.5 S)
c(s20,w) / arb. unit
s20,w / S
p1
p2
p3
p4
p5
(c)
(d)
(e)
Integral scattering intensity / cm-1
Absorbance
Rg / Å
Elution volume / mL

Supplementary Figure 2. Characterization of ABC complex by SEC and AUC. (a) Sodium dodecyl sulfate polyacrylamide gel electrophoresis (SDS-PAGE) analysis of size exclusion chromatography (SEC) major fraction of KaiC (lane 1~3), and the mixtures of KaiA, KaiB and KaiC at 0: 9: 6 (lane 4~6), 2: 12: 6 (lane 7~9), 4: 12: 6 (lane 10~12), 6: 12: 6 (lane 13~15), 12: 12: 6 (lane 16~18), 18: 12: 6 (lane 19~21), and 24: 12: 6 (lane 22~24) under constant concentration of 25 μM KaiC (n=3 technical replicates) on 15% gels stained with Coomassie brilliant blue. B_6C_6 complex and $A_{12}B_6C_6$ complex were purified by SEC on Superose 6 increase 10/300 GL (see Materials and methods). The uncropped and unedited blot/gel image was provided as Supplementary Fig. S9. (b)AUC profile of Kai solutions. Pink, green, blue, red and black lines show the AUC profiles of KaiA, KaiB, KaiC, binary mixture (KaiB and KaiC), and ternary mixture (KaiA, KaiB and KaiC) solutions, respectively. The distinct peaks at 2.9 S, 3.6 S, 11.4 S, and 12.4 S on the former four solutions, indicated with broken lines, were assigned to KaiA dimer (A_2), KaiB tetramer (B_4), KaiC hexamer (C_6) and BC complex with 6:6 (B_6C_6), respectively (Supplementary Table 1). In the ternary mixture solution, the distinct and sharp peak at 17.5 S was clearly assigned to $A_{12}B_6C_6$ complex form its molecular weight as described in Supplementary Table 2. In addition, the other peaks in the profile of the ternary mixture solution indicated that the solution was not mono-dispersed: Peaks around $s_{20,w}$ =3.5, 14.5, and 19.7 S correspond to the mixture of A_2 and B_4, the other minor complex with a molecular mass of c.a. 610 kDa, and aggregated object, respectively (See Supplementary Table 2). (c-e) Coincidence display between elution from SEC system, observed scattering intensities, and R_g of (c) SAXS of $hA_{12}hB_6hC_6$ and iCM-SANS of (d) $hA_{12}hB_6hC_6$ and (e) $hA_{12}dB_6dC_6$ complexes. In upper panels, a green line indicates an elution profile of the ternary mixture solution (UV absorbance at 280 nm for panel (c) and at 260 nm for panels (d) and (e)) and open circles indicate the time evolution of the integrated intensities of the scattering profiles in the Q-range from 0.008 Å^{-1} to 0.015 Å^{-1}. In bottom panels, red circles show the time evolution of R_g. The scattering data for the analyses were obtained by averaging the time-resolved profiles over the pink-shaded zones. Error bars in panels (c)-(e) represent standard deviation of the mean.

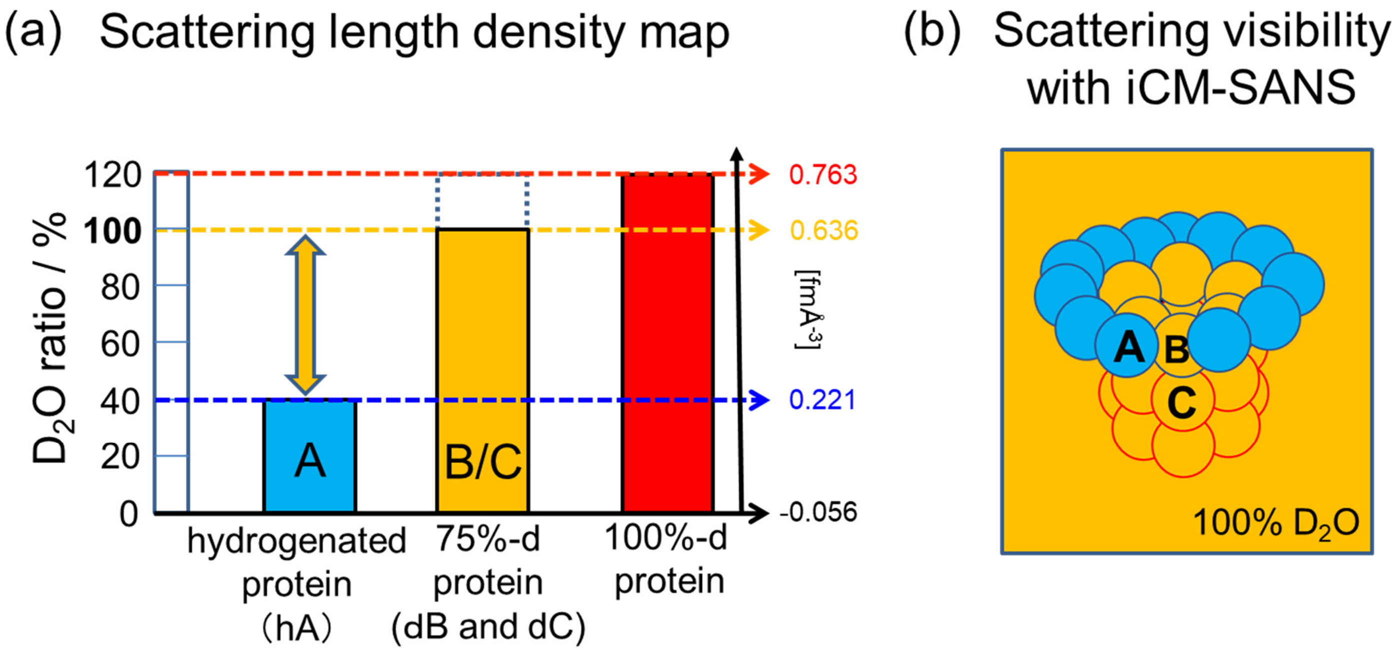

Supplementary Figure 3. Inverse Contrast Matching Small-Angle Neutron Scattering (iCM-SANS). (a) Neutron scattering length density map. Neutron scattering length densities of hydrogenated, 75%-deuterated and 100%-deuterated proteins are same as those of 40%, 100% and 120% D_2O solvents, respectively. It should be noted that there is no 120% D_2O solvent in reality, meaning that SANS cannot make 100%-deuterated protein invisible with any water solvent. (b) A schematic view of strategy of selective observation of KaiA protomers in ABC complex. When KaiA, KaiB and KaiC are hydrogenated, 75%-deuterated and 75%-deuterated, respectively, only KaiA is visible in the ABC complex in 100% D_2O.

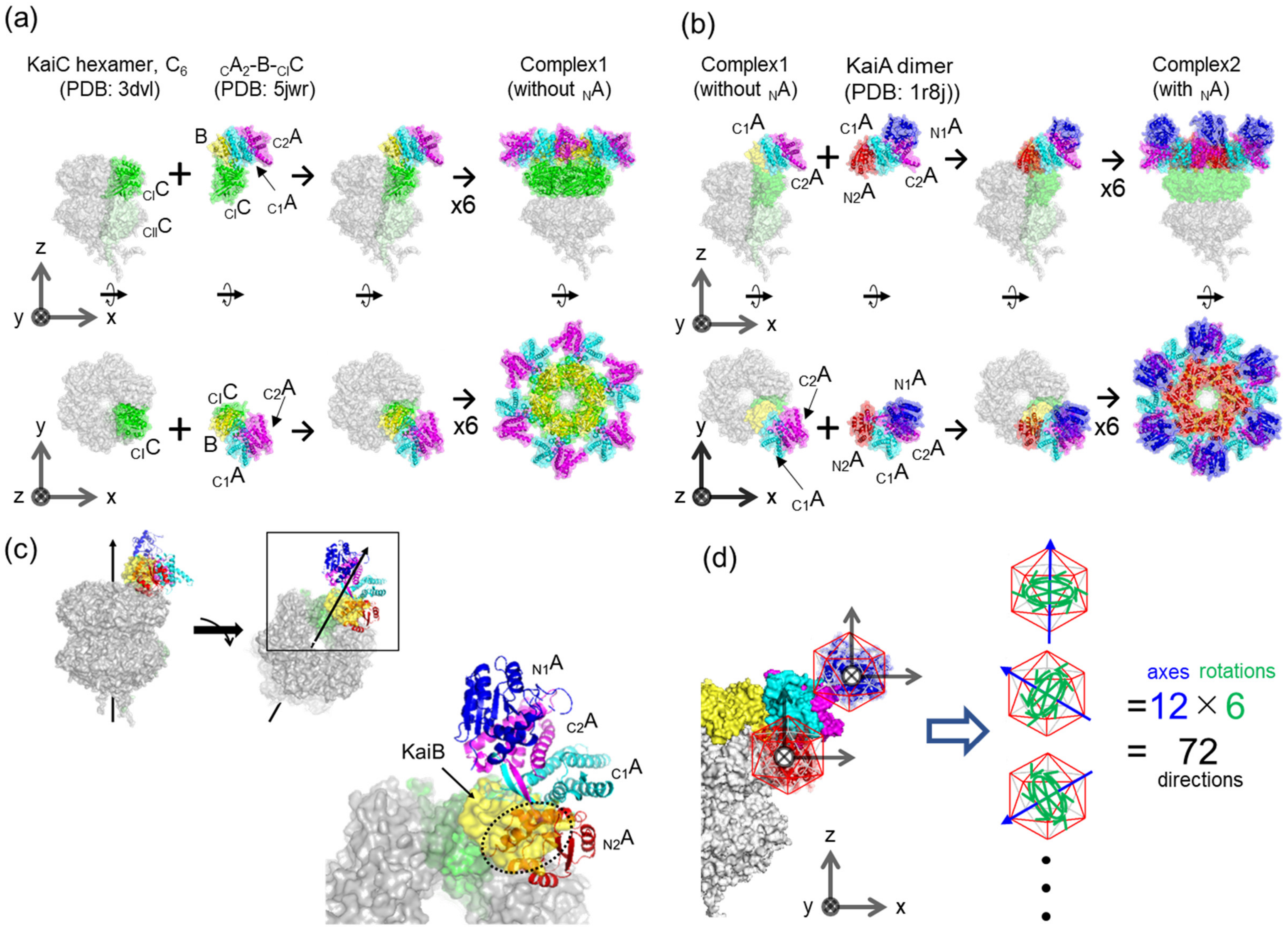

(a)
KaiC hexamer, C6
(PDB: 3dvl)
cA2-B-CIC
(PDB: 5jwr)
Complex1
(without NA)
B
C2A
C1A
CIC
CIIC
x6
z
y
x
(b)
Complex1
(without NA)
KaiA dimer
(PDB: 1r8j))
Complex2
(with NA)
C1A
N1A
C2A
N2A
(c)
KaiB
N1A
C2A
C1A
N2A
(d)
axes
rotations
=12 × 6
= 72
directions

Supplementary Figure 4. Modeling of $A_{12}B_6C_6$ complex. (a) Building scheme of Complex 1 with C_6 (PDB code: 3dvl) and six of $_{C}A_2$-B-$_{CI}$C (PDB code: 5jwr). The Complex 1 model was built by superposing the $_{C1}$C domains of C_6 on that of $_{C}A_2$-B-$_{CI}$C. Complex 1 well agreed to the cryo-EM structure (RMSD 3.9 Å). (b) Building scheme of Complex 2 with Complex 1 and six A_2 dimers (PDB code: 1r8j). The Complex 2 model was built by superposing the $_{C1}$A and $_{C2}$A domains of Complex 1 on those of the A_2 dimers. Inset shows connection of the three components, KaiC monomer in C_6 (PDB code: 3dvl), $_{C}A_2$-B-$_{CI}$C (PDB code: 5jwr) and full-length A_2 dimer (PDB code: 1r8j) in Complex 2. (c) Structural overlap between KaiB (yellow) and $_{N2}$A domain (red) in Complex 2. (left) Side view, (center) declined view, and (right) its enlarged view. (d) The overlapped zone is highlighted with a broken circle. (left) Translation scheme. Individual $_{N}$A domains (blue and red) were systematically moved at the interval of 3 Å along the (x, y, z)-axes. (right) Rotation scheme. The axis of $_{N}$A domain, which is defined locally on each domain, is set on one of 12 axes connecting the vertices of regular icosahedron (expressed by red and grey lines) to its center and the domain is rotated around the axis by one of six azimuth angels, Azimuth angles are $n\theta$ (θ = 60º, n = 0~5).

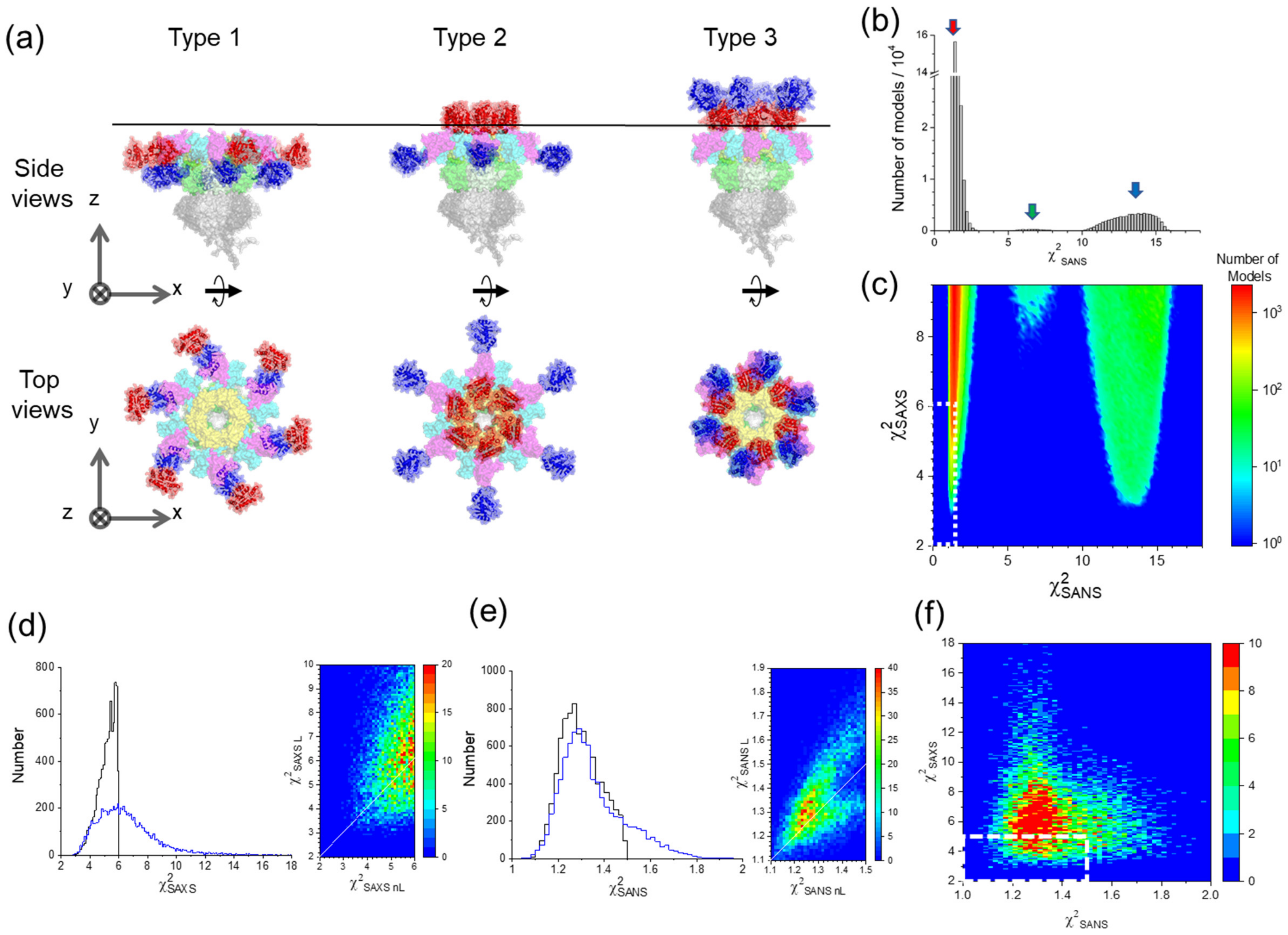

(a)
Type 1
Type 2
Type 3
Side views
Top views
(b)
Number of models / 10^4
χ² SANS
(c)
Number of Models
χ²SAXS
χ²SANS
(d)
Number
χ²SAXS
χ² SAXS L
χ² SAXS nL
(e)
Number
χ²SANS
χ² SANS L
χ² SANS nL
(f)
χ² SAXS
χ² SANS

Supplementary Figure 5. Estimation of structural models of $A_{12}B_6C_6$ complex by SAXS and SANS data. (a) Side and top views of three typical structures which well reproduce the experimental SAXS profile. The horizontal black line in the side views denotes the top plane of the hexameric KaiB ring. Type 1 has both $_{N1}$A (blue) and $_{N2}$A (red) domains below the reference plane. Type 2 has one of $_{N}$A domains below the reference plane and the other upper. Type 3 has both $_{N1}$A and $_{N2}$A domains upper the reference plane. (b) Number of SAXS-screened structural model, Types 1-3 as a function of χ^2_{SANS}. Three types separately distribute depending upon χ^2_{SANS}: Red, green, and blue arrows indicate distributions of Type 1, Type 2, and Type 3, respectively. (c) Correlation map of number distribution for χ^2_{SAXS} and χ^2_{SANS}. (d)-(f) Change of χ^2 for the structures with $\chi^2_{\mathrm{SAXS}} < 6.0$ and $\chi^2_{\mathrm{SANS}} < 1.5$ after linker addition. (d) Number of modes with linker (blue line) and without linker (black line) plotted against χ^2_{SAXS} (left). χ^2_{SAXS}-correlation between models with and without the linker (right). $\chi^2_{\mathrm{SAXS\,L}}$ (vertical) and $\chi^2_{\mathrm{SAXS\,nL}}$ (horizontal) indicate χ^2_{SAXS} with and without linker, respectively. The structures below the white line reduced χ^2_{SAXS} by linker addition. (e) Number of modes with linker (blue line) and without linker (black line) plotted against χ^2_{SANS} (left). and χ^2_{SANS}-correlation between models with and without the linker (right). $\chi^2_{\mathrm{SANS\,L}}$ (vertical) and $\chi^2_{\mathrm{SANS\,nL}}$ (horizontal) indicate χ^2_{SANS} with and without linker, respectively. The structures below the white line reduced χ^2_{SANS} by linker addition. (f) Distributions of models against their χ^2_{SAXS} and χ^2_{SANS}.

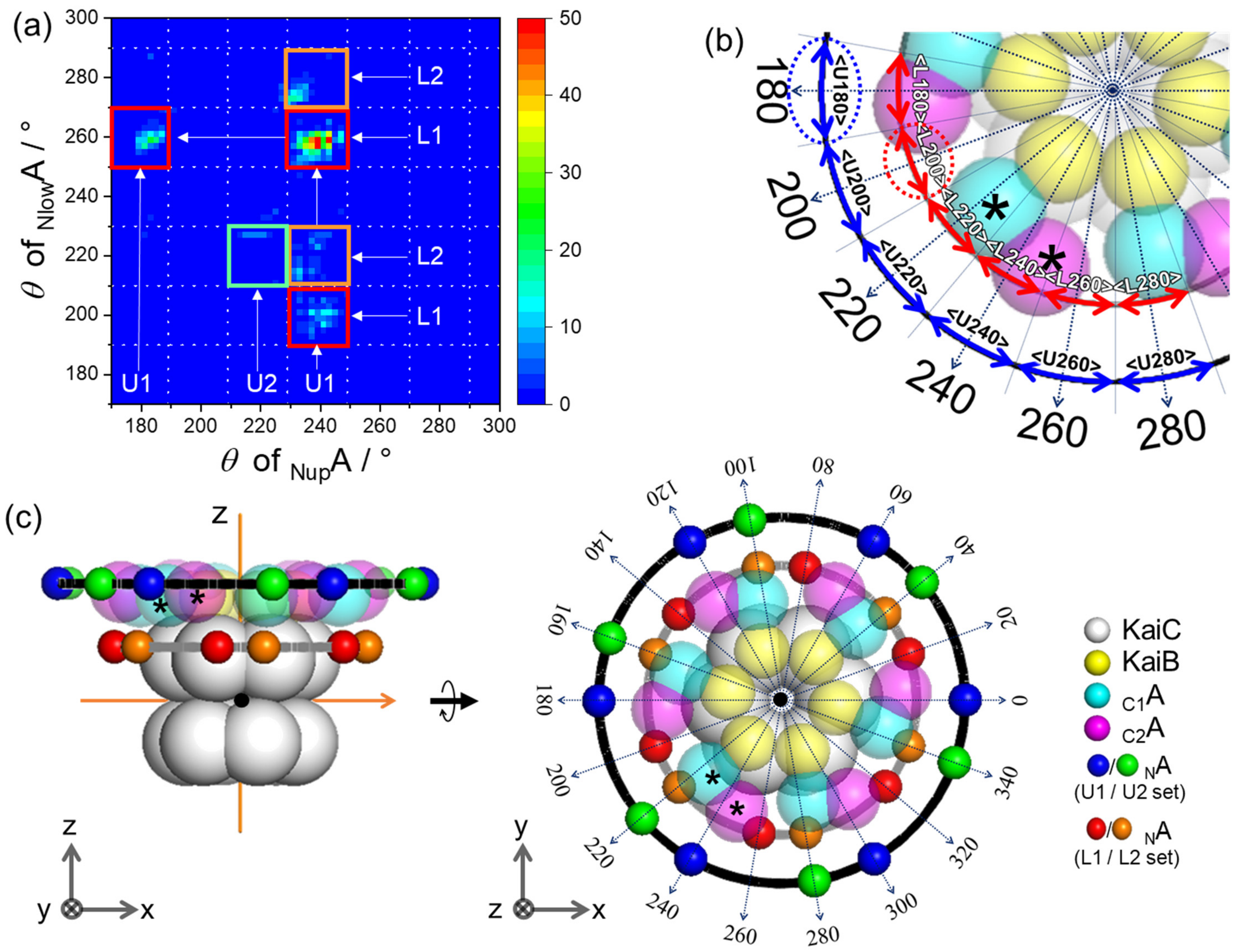

(a)
(b)
(c)
θ of NlowA / °
θ of NupA / °
L2
L1
U1
U2
KaiC
KaiB
C1A
C2A
NA
(U1 / U2 set)
(L1 / L2 set)

Supplementary Figure 6. Grouping of structural models of $A_{12}B_6C_6$ complex. (a) Distribution map of $_{N}$A domains connecting to $_{C1}$A (θ = 225º) and $_{C2}$A (θ = 250º) domains along the U and L rings. The cell positions are indicated with white arrows. Red, orange, and green squares show U1-L1, U1-L2 and U2-L2 cell combinations. (b) The cell positions on the U and L rings. Each position is denoted by the notation with the ring it belongs to and its angel as <ring+angle>. <U180> and <L200> are marked with blue and red dashed circles, respectively. (c) The whole distributions of $_{N}$A domains along the upper and lower rings. Blue, green, red, and orange spheres denote U1, U2, L1, and L2 cell sets, respectively.

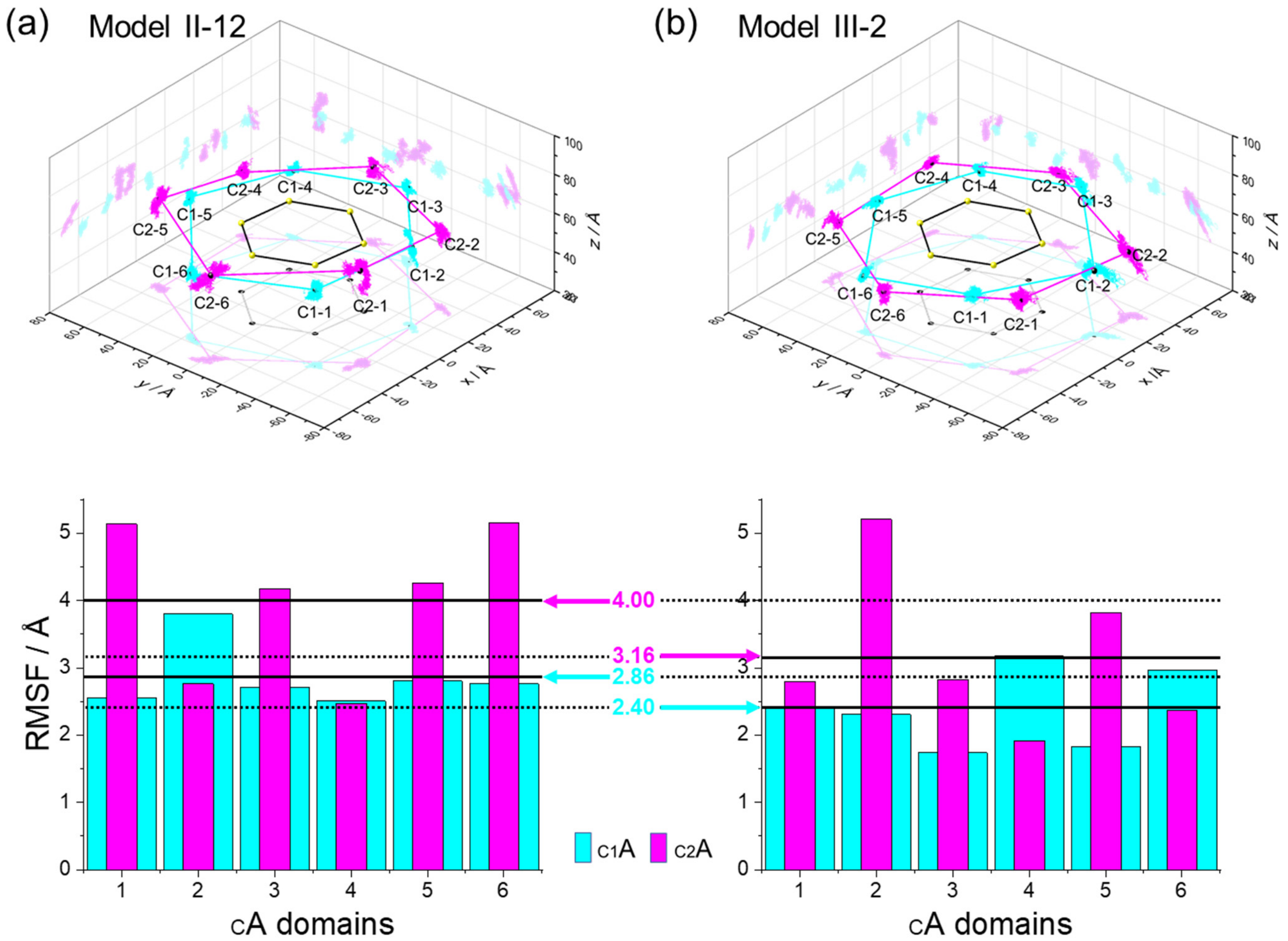

Supplementary Figure 7. Dynamical fluctuations of $_{C}$A domains in 100-ns-MD simulations. Trajectories (upper panes) and RMSFs (lower panels) of COM of $_{C}$A domains of Models II-12 (a) and III-2 (b). Cyan, magenta, and yellow colors represent the trajectories and RMSFs of $_{C1}$A, $_{C2}$A and KaiB domains, respectively. In the lower panels, the straight lines and numbers denote the average values of RMSFs.

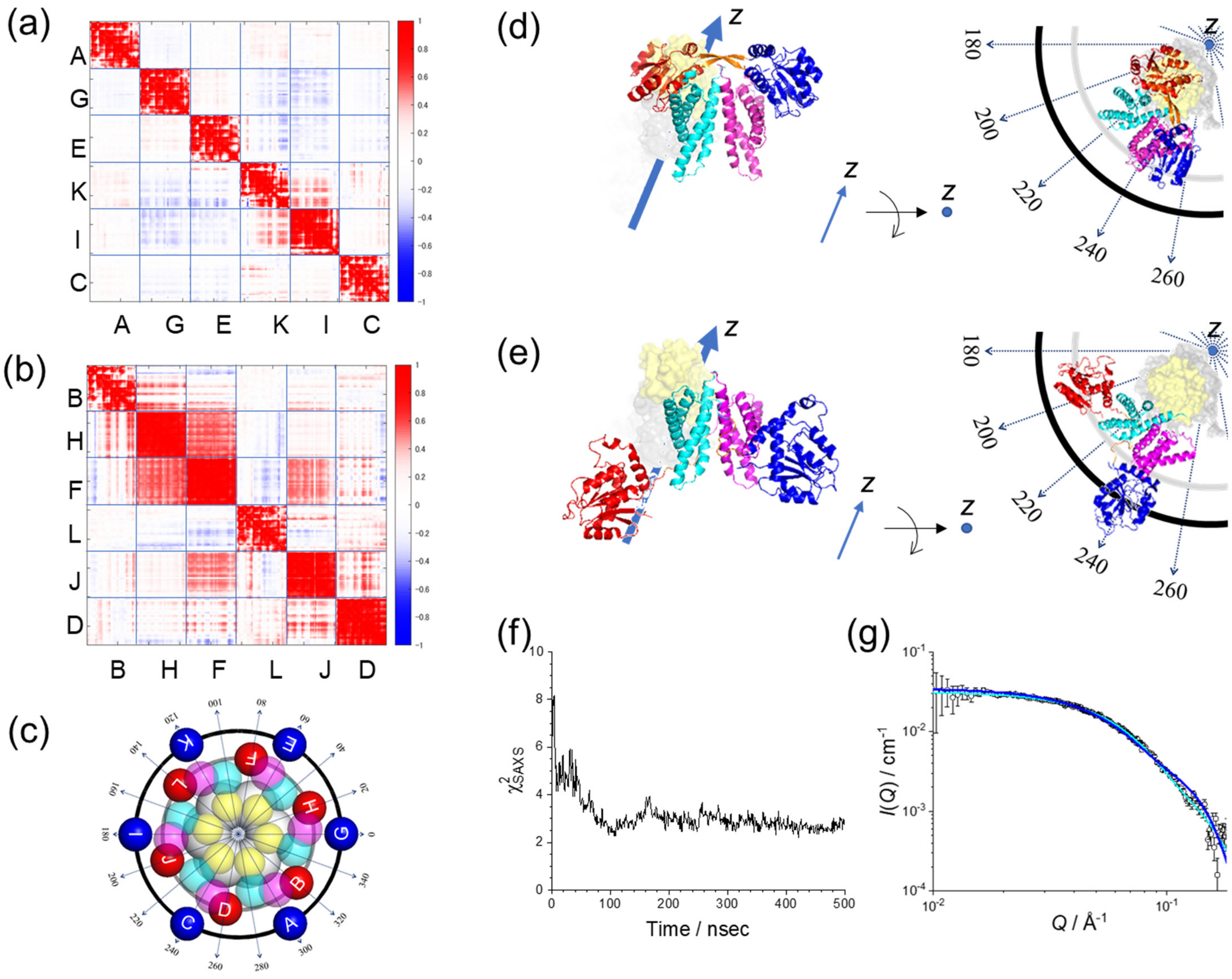
(a)
A
G
E
K
I
C
(b)
B
H
F
L
J
D
(c)
(d)
z
180
200
220
240
260
(e)
(f)
χ^2_{SAXS}
Time / nsec
(g)
I(Q) / cm^{-1}
Q / Å^{-1}

Supplementary Figure 8. Motion and conformation of $_{\mathrm{N}}$A domains in Model III-2. (a)-(b) Dynamical cross correlation maps of $_{\mathrm{N}}$A domains in Model III-2. (a) $_{\mathrm{N1}}$A domains (on the U ring), (b) $_{\mathrm{N2}}$A domains (on the L ring). Alphabets in the vertical and horizontal axes indicate the chain codes of N-terminal domains of KaiA. (c) Map of chain codes of $_{\mathrm{N}}$A domains as a guide for panels (a) and (b). (d) Crystal structure of A_2 (PDB code: 1r8j) superimposed onto Complex 1 (cryo-EM structure). (left) facing view and (right) top view from *z*-axis. Yellow and grey domains denote a KaiB protomer and a KaiC protomer, respectively. (e) Structure of A_2 in Model III-2. (left) facing view and (right) top view from *z*-axis. Yellow and grey domains denote a KaiB protomer and a KaiC protomer, respectively. (f) Time evolution of χ^2_{SAXS} of A_2 in the MD simulation. (g) Experimentally obtained (white circles), MD structure-based (blue line) and the crystal structure-based (cyan line) SAXS profiles. Error bars in panel (g) represent standard deviation of the mean.

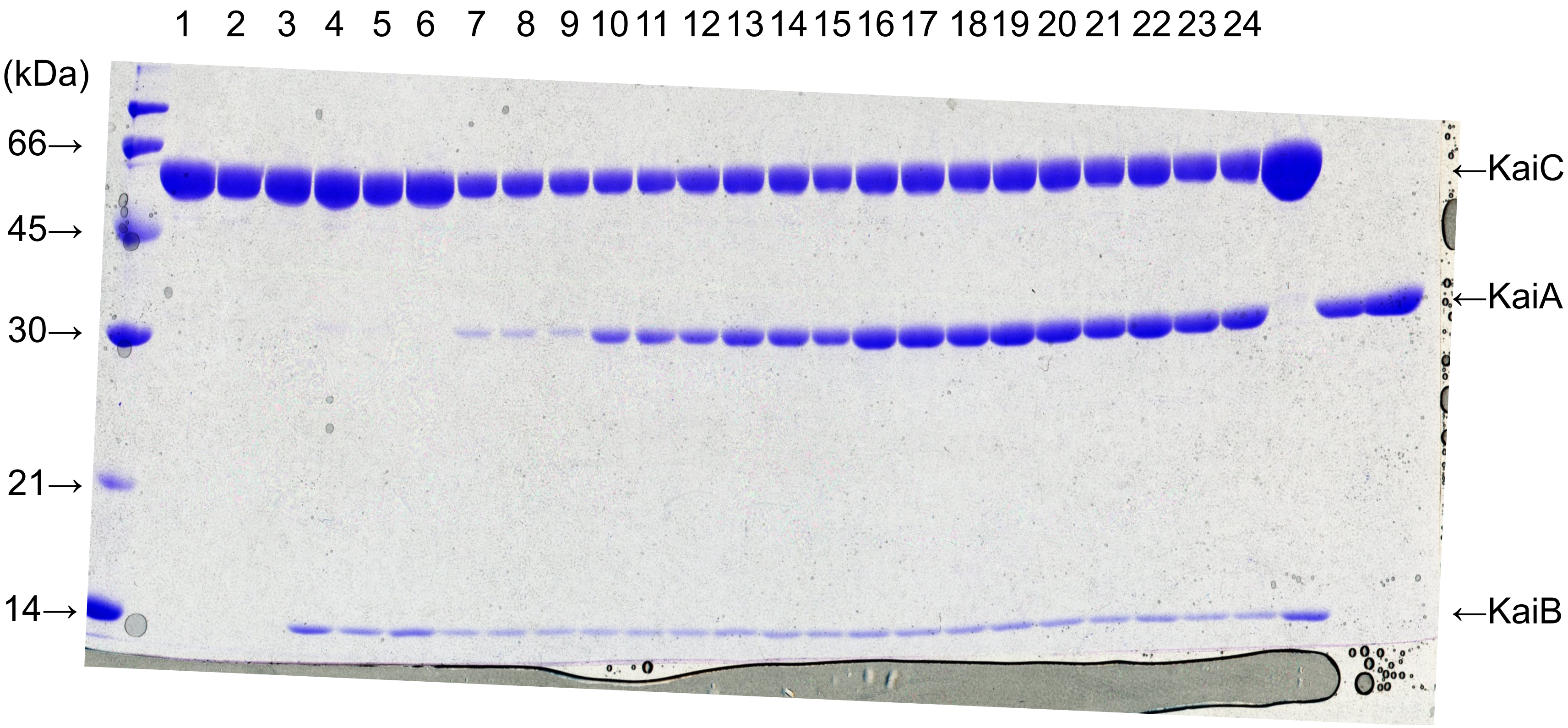

Supplementary Figure 9. Uncropped and unedited SDS-PAGE image of SEC fraction of ABC complex.

Supplementary notes

Supplementary note 1. Calculation of χ^2 for SAXS and SANS profiles

To select appropriate atomic models that well reproduced the experimentally obtained scattering profiles, we used χ^2 value defined as

$$\chi^2 = \frac{1}{N_p}\sum_{i=1}^{N_p}\left(\frac{I_e(Q_i) - cI(Q_i) + a}{\sigma(Q_i)}\right)^2, \text{(S1)}$$

where N_p was the number of experimental points Q_i; $I_e(Q_i)$, and $I(Q_i)$ were the experimental profile and that computed from an atomic model, respectively; $\sigma(Q_i)$ was the experimental error; c was a scale factor given by

$$c = \sum_{i=1}^{N_p} \frac{I_e(Q_i) I(Q_i)}{\sigma(Q_i)^2} / \sum_{i=1}^{N_p} \frac{I(Q_i)^2}{\sigma(Q_i)^2}; \text{(S2)}$$

and "a" was the offset that accounts for possible systematic errors due to mismatched buffers in the experimental data. The profiles were computed using CRYSOL for SAXS profiles[supplementary 1] and CRYSON for SANS profiles[supplementary 2]. Smaller χ^2 values indicated a better fit to the experimental profile but the χ^2 value less than 1.0 means the over fitting.

Supplementary note 2. Initial modeling of $A_{12}B_6C_6$ complex

We initially constructed the $A_{12}B_6C_6$ complex according to the procedure shown in Supplementary Fig. 4a. In the figure, CI and CII domains ($_{CI}C$ and $_{CII}C$) in one of KaiC protomers are highlighted with green and light green, respectively, and the others with grey. The ternary complex $_{C}A_2$-B-$_{CI}C$ (PDB code: 5jwr) is also shown in Supplementary Fig. 4a: $_{C1}A$ (cyan) and $_{C2}A$ (magenta) domains are C-terminal domains of one A_2 dimer bound and unbound to KaiB, respectively, and KaiB is colored yellow, and $_{CI}C$ green. We first added the six of cA_2-B-c$_I$C to C_6 (PDB code: 3dvl) by superposing the $_{CI}C$ domain (green) and then obtained $A_{12}B_6C_6$ complex (Complex 1). The root-mean-square-deviation (RMSD) of heavy atoms between the two $_{CI}C$ domains from 3dvl and 5jwr is about 2.5 Å.

Next, as shown in Supplementary Fig. 4b, we placed six full-length A_2 dimers onto Complex 1 by superposing the $_{C1}A$ and $_{C2}A$ domains: $_{N1}A$ (blue) and $_{N2}A$ (red) domains are N-terminal domains of A_2 linking to c$_1$A (cyan) and c$_2$A (magenta) domains, respectively. We named this overall structure model of $A_{12}B_6C_6$ complex as Complex 2. The RMSD between two sets of superimposed $_{C}A$ portions from the two crystal structures (PDB codes: 5jwr and 1r8j) was about 2.4 Å, suggesting that the quaternary structure of dimeric $_{C}A$ domains is maintained upon binding to KaiB.

There was a problem in Complex 2 that $_{N2}$A domain (red) heavily overlapped with KaiB (yellow) as shown in Supplementary Fig. 4c, indicating that Complex 2 is not a real structure. The SAXS profile and its Guinier plot of Complex 2 are shown in Fig.1a,b with cyan color, which also indicate discrepancy with the experimental curve.

Supplementary note 3. Inverse Contrast Matching Small-Angle Neutron Scattering (iCM-SANS)

Neutron scattering intensity is proportion to the square of the difference in scattering length density between solute and solvent. Therefore, 75%-deuterated protein is invisible but hydrogenated one is visible in 100% D_2O solvent (Supplementary Fig. 2). In the case of the complex consisting of hydrogenated domain/subunit and 75%-deuterated ones in 100% D_2O, SANS allows us to only observes the hydrogenated domain/subunit, this is named as inverse contrast matching SANS (iCM-SANS)[supplementary 3-7].

Supplementary note 4. Modeling of $A_{12}B_6C_6$ complex without any atomic overlap

In this step, we assumed that the six A_2 dimers in $A_{12}B_6C_6$ complex adopted the same conformations. This assumption saved computational cost and only two $_N$A domains in one A_2 were moved independently. This assumption was liberated in the final step, in which the structural stability was tested by MD simulation. Each N-terminal domain was systematically moved at the interval of 3 Å along the (x, y, z)-axes starting from the position in Complex 2 (Supplementary Fig. 4d) and was given 72 different orientations (the minimum angular difference between two orientations was about 60 degree: Supplementary Fig. 4d). In this treatment, we regarded the regions from V1 to Q161 in KaiA proteins as N-terminal domains ($_N$A domains) and those from E162 to N181 as linker regions which were not included in this step of the modeling. Instead, the Cα distance between Q161 (the end of $_N$A domain) and L182 (the beginning of C-terminal domain, $_C$A domain) in KaiA was limited less than 60 Å, which is a distance that can be connected with a linker. The linkers were modeled later at step 4. Finally, we obtained 20 million of the models of $A_{12}B_6C_6$ complex which did not have any atomic clash.

Supplementary note 5. Grouping of structural models of $A_{12}B_6C_6$ complex

We examined the 1,550 model structures within the white dotted box in Supplementary Fig. 5f. The spatial coordinates are set for describing C_6 sub-complex: an origin and *z* axis are a center of mass (COM) and a six-fold symmetry axis of C_6 sub-complex, respectively, and *x* and *y*-axes are set as shown in Fig. 3a and Supplementary Fig. 6c.

Firstly, we investigated the locational distribution of the COMs of $_{N}A$ domains. As shown in Fig. 3b, the COMs clearly distributed on two rings, named as the upper (U) and the lower (L) rings: the averages of radius *r* and height *z* of the U ring were $(r_1, z_1) = (91, 72)$ and those of the L ring were $(r_2, z_2) = (72, 38)$ in unit of Å. Fig. 3a also shows the relative positions of the two rings for Complex1, of which components are expressed with color spheres.

Next, we investigated the distribution of $_{N}A$ domains along the two rings. Supplementary Fig. 6a shows the correlational distributions map of two $_{N}A$ domains in one A_2 dimer, of which $_{C1}A$ (θ=225°) and $_{C2}A$ (θ=250°) are marked with asterisks in Supplementary Fig. 6b,c: θ corresponds to azimuth angle on x-y plane. It should be noted that the $_{N}A$ domains formed clusters in the map. To clarify the feature of cluster distribution, we divided the regions along the two rings into cells with 20 degrees width and introduced the notation which indicated

the cell with its ring and angel as <ring+angle> shown in Supplementary Fig. 6b: for examples, <U180> and <L200> indicate the cell regions on the U ring at 180° ± 10º (surrounded by blue dashed circle in Supplementary Fig. 6b) and on the L ring at 200° ± 10º (surrounded by red dashed circle in Supplementary Fig. 6b), respectively. Utilizing this notation, along the U ring, $_{\mathrm{N}}$A domains belonging to the one A_2 dimer ($_{\mathrm{C1}}$A (θ=225º) and $_{\mathrm{C2}}$A (θ=250º)) were frequently located on three cells, <U180>, <U220> and <U240> as shown in Supplementary Fig. 6a. Considering the 6-fold-symetry, <U180> and <U240> are structurally equivalent cells. Accordingly, it was revealed that, along the full U ring, the $_{\mathrm{N}}$A domains were distributed to two exclusive cell-sets, U1- and U2-sets: U1-set consists of six cells, <U000>, <U060>, <U120>, <U180>, <U240> and <U300> expressed with blue spheres, and U2-set consists of six cells, <U040>, <U100>, <U160>, <U220>, <U280> and <U340> expressed with green spheres (Supplementary Fig. 6c). With the same manner, along the full L ring, the $_{\mathrm{N}}$A domains were also distributed to two exclusive cell-sets, L1- and L2-sets: L1-set consists of six cells, <L020>, <L080>, <L140>, <L200>, <L260> and <L320> expressed with red spheres, and L2-set consists of six cells, <L040>, <L100>, <L160>, <L220>, <L280> and <L340> expressed with orange spheres (Supplementary Fig. 6c).

As mentioned above, two $_{\mathrm{N}}$A domains in one A_2 protomer are located on the U and the L rings in a mutually exclusive manner.

Supplementary Fig. 6a also shows the correlated distributions of $_{N}$A domains between the U and the L rings. There were three pair distributions, U1-L1 (red squares), U1-L2 (orange squares) and U2-L2 (a green square), but no U2-L1 pair. Furthermore, considering the linker connections from $_{N1}$A to $_{C1}$A and from $_{N2}$A to $_{C2}$A, we classified all structural models of KaiABC complex into eight groups. Fig. 3c,d shows the correlation distribution map of $_{N1}$A and $_{N2}$A domains which are connected to $_{C1}$A (θ=225°) and $_{C2}$A (θ=250°), respectively, indicating the locational correlations of the eight groups. The models were classified into eight groups, i.e. Groups I-VI plus Groups III' and V'. In Groups I-VI, the $_{N1}$A and $_{N2}$A domains are located on the U and L rings, respectively (Fig. 3c). In contrast, in Groups III' and V', the $_{N1}$A and $_{N2}$A domains are located on L and U rings, respectively (Fig. 3d). Fig. 3e-l show the structural features of the eight groups in detail. As shown in Fig. 3e-h, Groups I-III and III' have $_{N1}$A and $_{N2}$A domains falling in U1-L1 combination sets. The difference between them is the linker connection between $_{N}$A and $_{C}$A domains, as highlighted by blue line surrounding one A_2 protomer: For example, the $_{N1}$A and $_{N2}$A domains in Group I were located at <U180> and <L260> cells, respectively (for other Groups, see Supplementary Table 3). Again, the locational correlations of $_{N1}$A and $_{N2}$A domains between Groups III and III' are opposite. As shown in Fig. 3i-k, Groups IV, V and V' have $_{N1}$A and $_{N2}$A domains falling in U1-L2 combination set. Their difference between is also the linker connection between $_{N}$A and $_{C}$A

domains, as highlighted by blue line surrounding one A_2 protomer. Again, the locational correlations of $_{N1}A$ and $_{N2}A$ domains between Groups V and V' are opposite. As shown in Fig. 3l, Group VI has $_{N1}A$ and $_{N2}A$ domains falling in U2-L2 combination sets. The structural features of the eight groups are summarized in Supplementary Table 3.

Supplementary note 6. Comparison of KaiA dimer conformations in crystal, complex and solution

We compared the A_2 structure in Model III-2 with the crystal structure of A_2 alone (PDB code: 1r8j) and found their considerable difference (Supplementary Fig. 7d,e). The crystal structure of A_2 well reproduced its SAXS profile in solution ($\chi^2_{\mathrm{SAXS}} = 1.6$), indicating that structure of A_2 in solution could be quite similar to the crystal structure. This raised a question whether A_2 in solution undergoes a conformational change upon complex formation. To address this question, we performed MD simulation of A_2 starting from the A_2 conformation in Model III-2. Supplementary Fig. 7f shows the time evolution of χ^2_{SAXS}. Unexpectedly, χ^2_{SAXS} was dropped to around 2.5 in a short time (~100 ns). Supplementary Fig. 7g compares the experimental SAXS profile with the theoretical profiles computed from the MD-derived model and the crystal structure. These results suggest that the A_2 potentially has multiple stable conformations and one of them could make induced fit when binding to the B_6C_6 complex.

Supplementary Table 1. Parameters of samples for AUC measurements.

Samples	c [mg·mL^{-1}]	$s_{20,w}$ [S]	f / f_0	M*[1] [kDa]	M*[2] [kDa]
KaiA	0.5	3.6	1.49	64	66 (A_2)
KaiB	0.5	2.9	1.52	45	47 (B_4)
KaiC	0.5	11.4	1.58	346	356 (C_6)
Binary Mixture ([B]:[C]=6:6)	0.6	12.4	1.55	411	427 (B_6C_6)
Ternary Mixture ([A]:[B]:[C]=24:12:6)	1.0	17.5	1.72	810	822 ($A_{12}B_6C_6$)

*1: Calculated from the AUC profile. *2: Calculated from the sequence.

Supplementary Table 2. Peak parameters in AUC profile of the ternary mixture (KaiA, KaiB and KaiC) solution.

Peak	$s_{20,w}$ [S]	f / f_0	M*[1][kDa]	r [%]	t [%]
p1	3.5	1.47	59	34.1	3.7
p2	5.2	1.47	107	1.9	0.4
p3	14.5	1.72	611	2.9	3.3
p4	17.5	1.72	810	58.9	88.6
p5	19.7	1.72	970	2.2	4.0

*1: Calculated from the AUC profile.

Supplementary Table 3: Structural features of the eight groups.

Group	I	II	III	III’
Cell combination	U1-L1	U1-L1	U1-L1	U1-L1
$_{N}$A combination (U-L)	$_{N1}$A- $_{N2}$A	$_{N1}$A- $_{N2}$A	$_{N1}$A- $_{N2}$A	$_{N2}$A- $_{N1}$A
A_2 protomer ($_{N1}$A- $_{N2}$A)	<U180>-<L260>	<U240>-<L260>	<U240>-<L200>	<L200>-<U240>
Group	IV	V	V’	VI
Cell combination	U1-L2	U1-L2	U1-L2	U2-L2
$_{N}$A combination (U-L)	$_{N1}$A- $_{N2}$A	$_{N1}$A- $_{N2}$A	$_{N2}$A- $_{N1}$A	$_{N1}$A- $_{N2}$A
A_2 protomer ($_{N1}$A- $_{N2}$A)	<U240>-<L280>	<U240>-<L220>	<L220>-<U240>	<U220>-<L220>

Supplementary Table 4. Details of the MD simulations

Model	Box size[*1] (nm)	Number of atoms of model	Number of water molecules (x1000)	Number of Na^{+} ions	Number of Cl^{-} ions
KaiABC complex (384 models)	26.7-30.2	109650	585-863	1954-2723	1718-2387
KaiA dimer (1 model)	12.1	9028	54	192	158

1*: Cubic box

Supplementary References

1 Svergun, D., Barberato, C. & Koch, M. H. CRYSOL–a program to evaluate X-ray solution scattering of biological macromolecules from atomic coordinates. *Journal of applied crystallography* **28**, 768-773 (1995).

2 Svergun, D. *et al.* Protein hydration in solution: experimental observation by x-ray and neutron scattering. *Proceedings of the National Academy of Sciences* **95**, 2267-2272 (1998).

3 Sugiyama, M. *et al.* Conformational characterization of a protein complex involving intrinsically disordered protein by small-angle neutron scattering using the inverse contrast matching method: a case study of interaction between α-synuclein and PbaB tetramer as a model chaperone. *Journal of Applied Crystallography* **47**, 430-435, doi:10.1107/s1600576713033475 (2014).

4 Sugiyama, M. *et al.* Structural characterization of the circadian clock protein complex composed of KaiB and KaiC by inverse contrast-matching small-angle neutron scattering. *Sci. Rep.* **6**, 35567, doi:10.1038/srep35567 (2016).

5 Yogo, R. *et al.* Characterization of conformational deformation-coupled interaction between immunoglobulin G1 Fc glycoprotein and a low-affinity Fcγ receptor by deuteration-assisted small-angle neutron scattering. *Biochemistry and biophysics reports* **12**, 1-4 (2017).

6 Bernadó, P., Shimizu, N., Zaccai, G., Kamikubo, H. & Sugiyama, M. Solution scattering approaches to dynamical ordering in biomolecular systems. *Biochim Biophys Acta Gen Subj* **1862**, 253-274, doi:10.1016/j.bbagen.2017.10.015 (2018).

7 Inoue, R. *et al.* Elucidation of the mechanism of subunit exchange in αB crystallin oligomers. *Sci. Rep.* **11**, 1-9 (2021).
