# Supplementary material for: Overall structure of fully assembled cyanobacterial KaiABC circadian clock complex by an integrated experimental-computational approach
Source: Commun Biol. 2022 Mar 10;5:184. doi: 10.1038/s42003-022-03143-z (PMC8913699; doi:10.1038/s42003-022-03143-z)
Supplement: Supplementary file 3 — Description of Additional Supplementary Files [file 42003_2022_3143_MOESM3_ESM.pdf]

Description of Additional Supplementary Files

File name: Supplementary Movie 1
Description: *Top view of Model III-2.* Top view of dynamic motion of Model III-2 in 100ns MD simulation. Please download a file “KaiABC_s_movie_1.mp4”.

File name: Supplementary Movie 2
Description: *Side view of Model III-2.* Side view of dynamic motion of Model III-2 in 100ns MD simulation. Please download a file “KaiABC_s_movie_2.mp4”.

File name: Supplementary Data 1
Description: Time evolutions of X^2 of SAXS of all models in 100 ns MD simulations.

File name: Supplementary Data 2
Description: Time evolutions of X^2 of SAXN of all models in 100 ns MD simulations.
